# Six 1-aroyl-4-(4-meth­oxy­phen­yl)piperazines: similar mol­ecular structures but different patterns of supra­molecular assembly

**DOI:** 10.1107/S2056989019010491

**Published:** 2019-07-26

**Authors:** Haruvegowda Kiran Kumar, Hemmige S. Yathirajan, Belakavadi K. Sagar, Sabine Foro, Christopher Glidewell

**Affiliations:** a Department of Studies in Chemistry, University of Mysore, Manasagangotri, Mysuru-570 006, India; b Department of Studies in Chemistry, University of Mysore, Manasagangotri, Mysore-570 006, India; c Institute of Materials Science, Darmstadt University of Technology, Petersenstrasse 23, D-64287 Darmstadt, Germany; d School of Chemistry, University of St Andrews, St Andrews, Fife KY16 9ST, Scotland

**Keywords:** piperazines, crystal structure, isomorphism, disorder, hydrogen bonding, supra­molecular assembly

## Abstract

Six new 1-aroyl-4-(4-meth­oxy­phen­yl)piperazines have been prepared, using coupling reactions between benzoic acids and *N*-(4-meth­oxy­phen­yl)piperazine. There are no significant hydrogen bonds in the structure of 1-benzoyl-4-(4-meth­oxy­phen­yl)piperazine, C_18_H_20_N_2_O_2_, (I). The mol­ecules of 1-(2-fluoro­benzo­yl)-4-(4-meth­oxy­phen­yl)piperazine, C_18_H_19_FN_2_O_2_, (II), are linked by two C—H⋯O hydrogen bonds to form chains of rings, which are linked into sheets by an aromatic π–π stacking inter­action. 1-(2-Chloro­benzo­yl)-4-(4-meth­oxy­phen­yl)piperazine, C_18_H_19_ClN_2_O_2_, (III), 1-(2-bromo­benzo­yl)-4-(4-meth­oxy­phen­yl)piperazine, C_18_H_19_BrN_2_O_2_, (IV), and 1-(2-iodo­benzo­yl)-4-(4-meth­oxyphen­yl)piperazine, C_18_H_19_IN_2_O_2_, (V), are isomorphous, but in (III) the aroyl ring is disordered over two sets of atomic sites having occupancies of 0.942 (2) and 0.058 (2). In each of (III)–(V), a combination of two C—H⋯π(arene) hydrogen bonds links the mol­ecules into sheets. A single O—H⋯O hydrogen bond links the mol­ecules of 1-(2-hy­droxy­benzo­yl)-4-(4-meth­oxy­phen­yl)piperazine, C_18_H_20_N_2_O_3_, (VI), into simple chains. Comparisons are made with the structures of some related compounds.

## Abstract 2

Six new 1-aroyl-4-(4-meth­oxy­phen­yl)piperazines have similar mol­ecular structures, but their supra­molecular assembly ranges from simple chains, *via* a chain of rings, to complex sheets.

## Chemical context   

**Figure 1:**
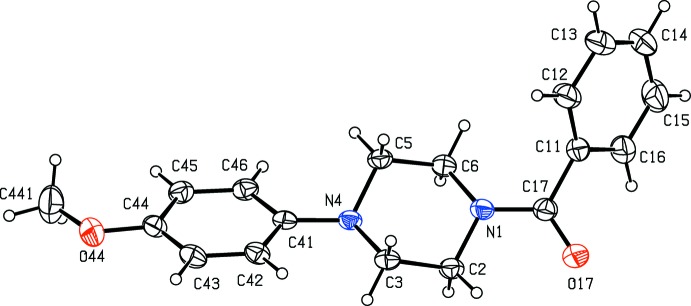
The mol­ecular structure of compound (I) showing the atom-labelling scheme. Displacement ellipsoids are drawn at the 30% probability level.

Piperazines are found in a wide range of compounds which are active across a number of different therapeutic areas such as anti­bacterial, anti­depressant, anti­fungal, anti­malarial, anti­psychotic, and anti­tumour activity (Brockunier *et al.*, 2004 ▸; Bogatcheva *et al.*, 2006 ▸), and a number of these areas have recently been reviewed (Elliott, 2011 ▸; Kharb *et al.*, 2012 ▸; Asif, 2015 ▸; Brito *et al.*, 2019 ▸). 1-(4-Meth­oxy­phen­yl)piperazine has been found to inhibit the re-uptake and accelerate the release of mono­amine neurotransmitters such as dopamine and serotonin, with a mechanism of action similar to that of recreational drugs such as amphetamines, but with significantly lower abuse potential (Nagai *et al.*, 2007 ▸). With these considerations in mind, we have now synthesized and characterized a series of closely related 1-aroyl-4-(4-meth­oxy­phen­yl)piperazines, using a straightforward coupling reaction between *N*-(4-meth­oxy­phen­yl)piperazine and a benzoic acid, promoted by 1-(3-di­methyl­amino­prop­yl)-3-ethyl­carbodimide as the dehydrating agent. Here we report the mol­ecular and supra­molecular structures of compounds (I)–(VI) (Figs. 1 ▸–6 ▸ ▸ ▸ ▸ ▸) which we compare with the structures of some related compounds. As well as these 2-substituted derivatives, we have also synthesized 1-(4-fluoro­benzo­yl)-4-(4-meth­oxy­phen­yl)piperazine (VII), but to date we have been unable to obtain any crystalline material suitable for single crystal X-ray diffraction.
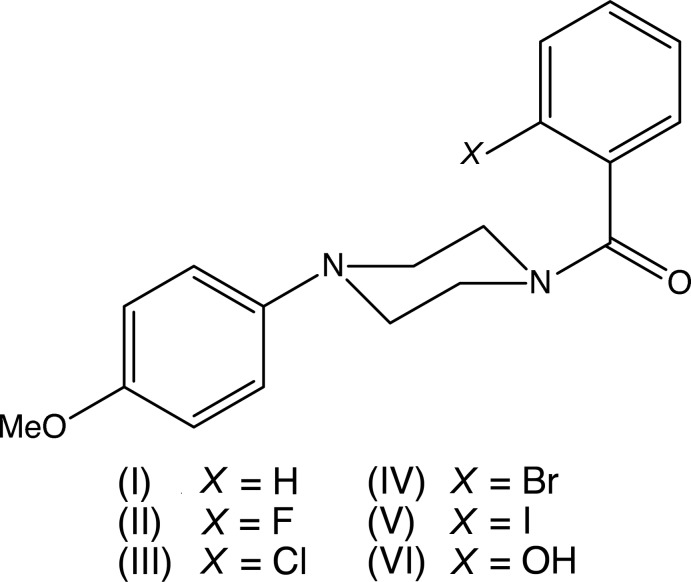


## Structural commentary   

**Figure 3:**
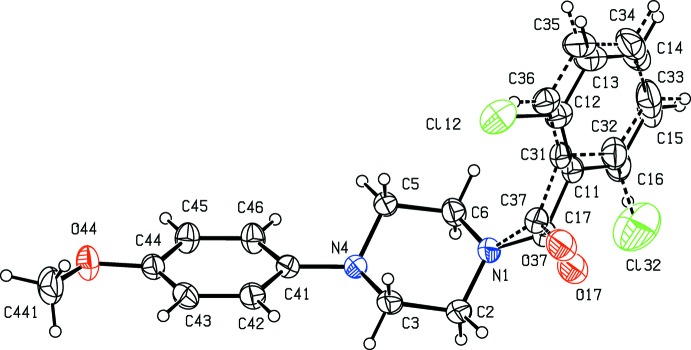
The mol­ecular structure of compound (III) showing the atom-labelling scheme, and the disorder of the 2-chloro­benzoyl unit. The major disorder component is drawn using full lines and the minor disorder component is drawn using broken lines. Displacement ellipsoids are drawn at the 30% probability level.

In the 2-chloro derivative (III), the benzoyl substituent is disordered over two sets of atomic sites having refined occupancies for the crystal selected for data collection of 0.942 (2) and 0.058 (2): in these two disorder forms, the chloro substituents occupy sites on opposite sides of the adjacent aryl ring (Fig. 3 ▸). Compounds (III), (IV) and (V) have similar unit-cell dimensions (Table 2 ▸) and, discounting the disorder in (III), each can be refined using the atomic coordinates of another as the starting point. However, these three structures exhibit several minor differences: firstly, the benzoyl group is disordered over two sets of atomic sites in (III), but not in (V); in (IV), the disorder was found to be very minor, *ca* 1.6%, such that attempted refinement of this small fraction was regarded as unrealistic and thus the ordered model was preferable. Secondly, there is a short inter­molecular I⋯O contact in (V), which has no Cl⋯O or Br⋯O analogue in (III) and (IV). Hence compounds (III)–(V) can be regarded as isomorphous, but not strictly isostructural (*cf*. Acosta *et al.*, 2009 ▸).

In each of the compounds reported here, the piperazine ring adopts an almost perfect chair conformation with the 4-meth­oxy­phenyl substituent occupying an equatorial site: the geometry at atom N1 is effectively planar and only in compound (I) is there a very slight pyramidalization at this site. For each compound, the reference mol­ecule was selected as one having a ring-puckering angle θ (Cremer & Pople, 1975 ▸) for the atom sequence (N1,C2,C3,N4,C5,C6) which was close to zero, as opposed to values close to 180° for the corresponding enanti­omers. In all of the compounds, the meth­oxy carbon atom C441 is very close to being coplanar with the adjacent aryl ring: the maximum displacement of this atom from the ring plane is 0.216 (16) Å in compound (V). Associated with this observation, we note that the two exocyclic O—C—C angles at atom C44 always exhibit differences in the range 8–10°: this behaviour is entirely consistent with the that previously observed in planar or nearly planar alk­oxy­arenes (Seip & Seip, 1973 ▸; Ferguson *et al.*, 1996 ▸). It is inter­esting to note that the meth­oxy group is oriented transoid to the carbonyl group in compounds (I) and (VI), but cisoid in compounds (II)–(V), suggesting that the methyl group may simply be acting in a space-filling role.

## Supra­molecular features   

The supra­molecular assembly in compounds (I)–(V) is dominated by contacts of C—H⋯O and C—H⋯π(arene) types (Table 1 ▸) and it is thus appropriate to define explicitly the criteria against which these contacts have been regarded as structurally significant hydrogen bonds. For single-atom acceptors, we adopt the distance criteria recommended in *PLATON* (Spek, 2009 ▸), based on the well-established concept of van der Waals radii (Bondi, 1964 ▸; Nyburg & Faerman, 1985 ▸; Rowland & Taylor, 1996 ▸), which provide an upper limit for H⋯O contacts of 2.60 Å, combined with the recommended (Wood *et al.*, 2009 ▸) lower limit of 140° for the *D*—H⋯*A* angle. For the C—H⋯π(arene) contacts in the isomorphous compounds (III)–(V), both the H⋯*Cg* distances and the C—H⋯*Cg* angles are entirely typical of C—H⋯π(arene) hydrogen bonds (Braga *et al.*, 1998 ▸). On this basis the C—H⋯O contacts in (II) can be regarded as significant, while the nearly linear C—H⋯O contacts in (III)–(V), which appear in each case to act cooperatively with a C–H⋯π hydrogen bond should be regarded as of marginal significance in (III) and (V).

**Figure 7:**
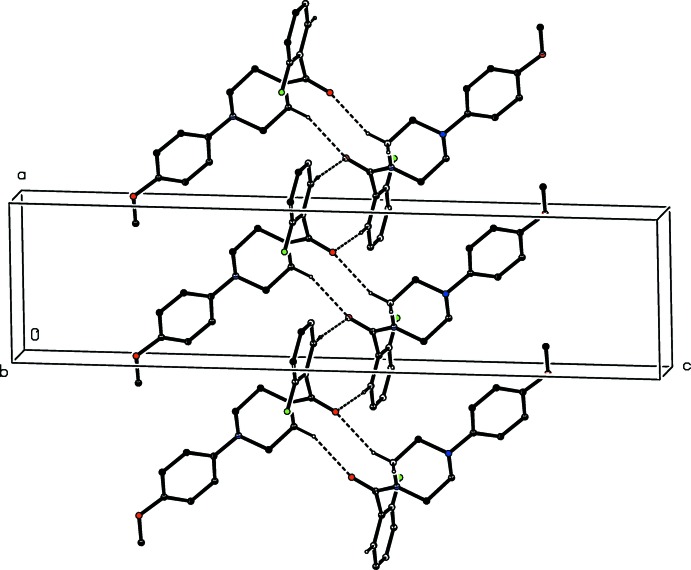
Part of the crystal structure of compound (II) showing the formation of a chain of rings running parallel to the [100] direction. Hydrogen bonds are shown as dashed lines and, for the sake of clarity, the H atoms bonded to those C atoms which are not involved in the motif shown have been omitted.

The sole direction-specific short inter­molecular contact in (I) is between mol­ecules related by a glide plane. The mol­ecules of compound (II) are linked by two independent C—H⋯O hydrogen bonds (Table 1 ▸) to form a chain of centrosymmetric rings in which 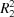
(10) (Etter, 1990 ▸; Etter *et al.*, 1990 ▸; Bernstein *et al.*, 1995 ▸) rings involving atom C2 as the donor and centred at (*n* + 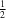, 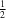, 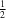) alternate with 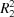
(10) rings involving atom C16 as the donor and centred at (*n*, 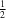, 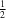), where *n* represents an integer in each case (Fig. 7 ▸). Chains of this type are linked into sheets by an aromatic π–π stacking inter­action: the fluorinated rings in the mol­ecules at (*x*, *y*, *z*) and (2 − *x*, 2 − *y*, 1 − *z*) are parallel with an inter­planar spacing of 3.520 (2) Å; the ring-centroid separation is 3.774 (2) Å and the ring-centroid offset is 1.360 (2) Å. This inter­action links the hydrogen-bonded chains into a sheet lying parallel to (001) in the domain 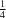 < *z* < 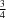: a second such sheet, related to the first by the translational symmetry operation, lies in the domain −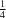 < *z* < 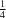, but there are no direction-specific inter­actions between adjacent sheets.

**Figure 8:**
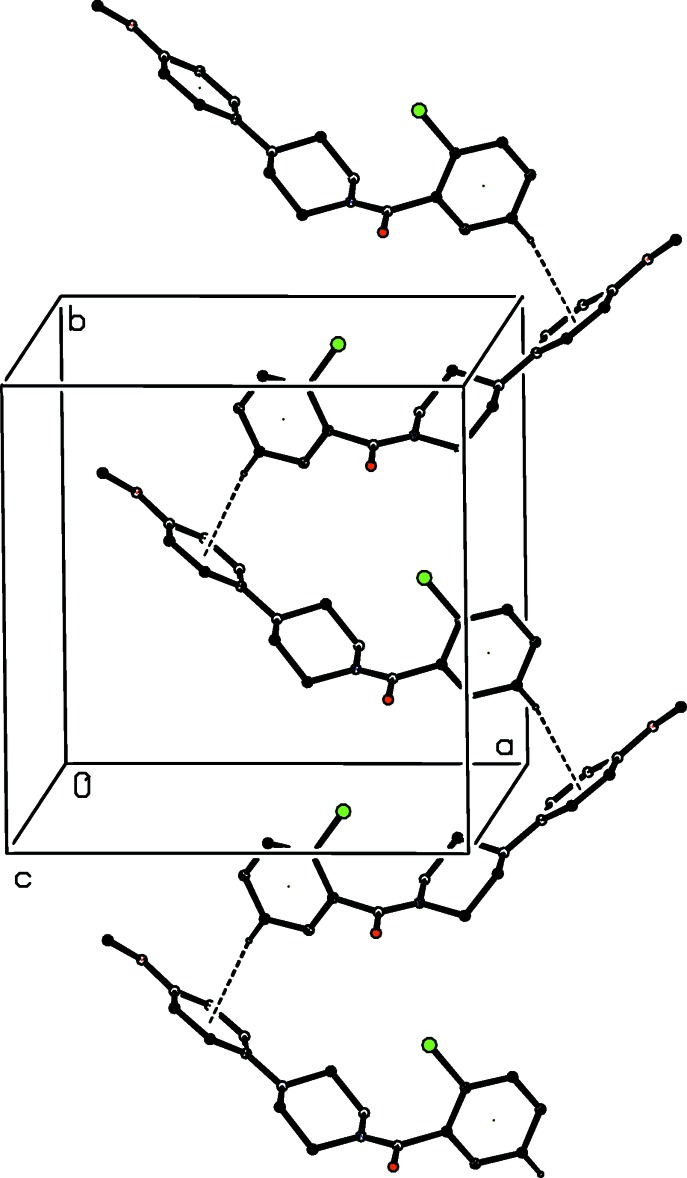
Part of the crystal structure of compound (III) showing the formation of a simple chain running parallel to the [010] direction. Hydrogen bonds are shown as dashed lines and, for the sake of clarity, the minor disorder component and the H atoms bonded to those C atoms which are not involved in the motif shown have been omitted.

**Figure 9:**
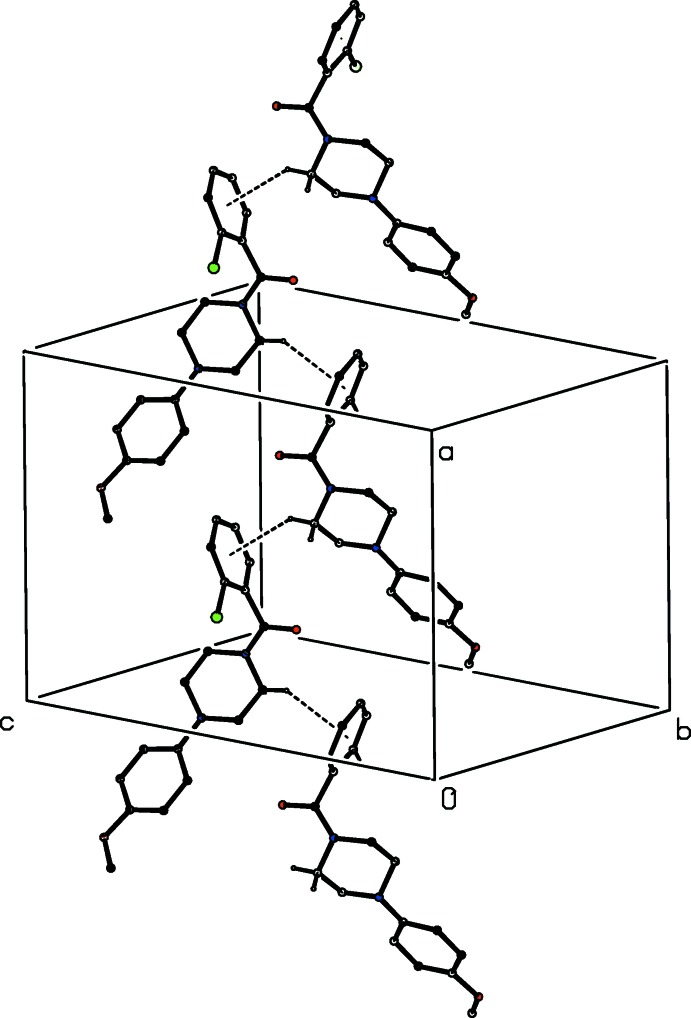
Part of the crystal structure of compound (III) showing the formation of a simple chain running parallel to the [100] direction. Hydrogen bonds are shown as dashed lines and, for the sake of clarity, the minor disorder component and the H atoms bonded to those C atoms which are not involved in the motif shown have been omitted.

As noted previously (see Section 2), the 2-chloro­benzoyl unit in compound (III) is disordered over two sets of atomic sites: however, the occupancy of the minor disorder component is low, and thus only the major component need be considered here. The supra­molecular assembly in each of (III)–(V) is essentially the same. A combination of two C—H⋯π(arene) hydrogen bonds, weakly augmented by a C—H⋯O Inter­action, links the mol­ecules into sheets, whose formation is readily analysed in terms of two one-dimensional sub-structures (Ferguson *et al.*, 1998*a* ▸,*b* ▸; Gregson *et al.*, 2000 ▸). In the simpler of the two sub-structures, mol­ecules related by the *b*-glide at *x* = 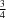 are linked by a C—H⋯π(arene) hydrogen bond to form a chain running parallel to the [010] direction (Fig. 8 ▸). In the second sub-structure, a C—H⋯π(arene) hydrogen bond links mol­ecules which are related by the 2_1_ screw axis along (*x*, 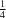, 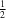) to form a chain running parallel to the [100] direction (Fig. 9 ▸). These two chain motifs combine to generate a sheet lying parallel to (001) in the domain 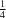 < *z* < 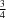. A second sheet, related to the first by inversion, lies in the domain 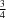 < *z* < 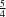, but there are no direction-specific inter­actions between adjacent sheets. However there is, in (V), a rather short inter­molecular I⋯O contact where I12⋯O17^i^ = 3.362 (7) Å and C12—I12⋯O17^i^ = 163.5 (2)° [symmetry code: (i) 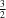 − *x*, 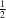 + *y*, *z*], as compared with the sum of van der Waals radii of 3.56 Å (Rowland & Taylor, 1996 ▸). This contact lies within the chain along [010] and so does not affect the overall two-dimensional nature of the supra­molecular assembly. However, short contacts of this type are not present in the structures of (III) and (IV), where the corresponding Cl⋯O and Br⋯O distances are 3.707 (4) and 3.708 (3) Å, respectively, as compared with the sums of van der Waals radii of 3.30 Å and 3.41 Å respectively. Simple considerations of electronegativity (Allen, 1989 ▸) indicate that in carbon–halogen bonds of type (ar­yl)C—*X*, the halogen atom carries a residual positive charge when *X* = I, but a residual negative charge when *X* = Cl or Br. On this basis (ar­yl)C—*X*⋯O=C inter­actions are expected to be attractive when *X* = I, but repulsive when *X* = Cl or Br, so accounting for the much shorter I⋯O distance in (V) as compared with the corres­ponding distances in (III) and (IV).

**Figure 10:**
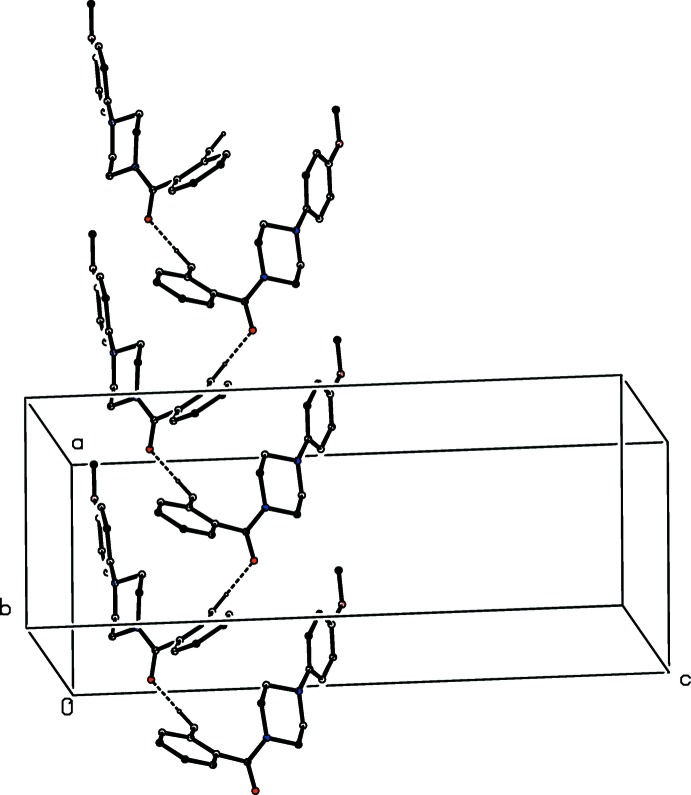
Part of the crystal structure of compound (VI) showing the formation of a *C*(6) chain running parallel to the [100] direction. Hydrogen bonds are shown as dashed lines and, for the sake of clarity, the H atoms bonded to C atoms have all been omitted.

The supra­molecular assembly in compound (VI) takes the form of simple *C*(6) chains running parallel to the [100] direction, in which mol­ecules related by the *a*-glide plane at *z* = 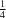 are linked by an O—H⋯O hydrogen bond (Table 1 ▸) (Fig. 10 ▸). A second chain of this type, related to the first by inversion, and two further chains related to the first pair by the *c*-glide planes, pass through each unit cell but there are no direction-specific inter­actions between adjacent chains.

Thus in summary, the supra­molecular assembly takes the form of a simple chain in compound (VI), a chain of rings in compound (II), and sheets in compounds (III), (IV) and (V).

## Database survey   

It is of inter­est briefly to compare the structures of compounds (I)–(VI) reported here with those of some closely related analogues. In 4-(4-meth­oxy­phen­yl)piperazin-1-ium chloride (Zia-ur-Rehman *et al.*, 2009 ▸), the ions are linked by two independent N—H⋯Cl hydrogen bonds: although the structure was described in the original report as dimeric, the ions are in fact linked into 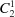
(4) chains. The mol­ecules of 1-acetyl-4-(4-hy­droxy­phen­yl)piperazine (Kavitha *et al.*, 2013 ▸) are linked by O—H⋯O hydrogen bonds to form simple *C*(12) chains, while those of 1-(2-iodo­benzo­yl)-4-(pyrimidin-2-yl)pip­erazine (Mahesha, Yathirajan *et al.*, 2019 ▸) are linked by a combination of C—H⋯O and C—H⋯π(arene) hydrogen bonds to form a three-dimensional framework structure which is further strengthened by both aromatic π–π stacking inter­actions and I⋯N halogen bonds. Finally, we note the structures of three closely related 1-(1,3-benzodioxolol-5-yl)methyl-4-(halobenzo­yl) piperazines (Mahesha, Sagar *et al.*, 2019 ▸), where the 3-fluoro­benzoyl derivative forms a three-dimensional framework structure built from C—H⋯O and C—H⋯π(arene) hydrogen bonds, whereas the structures of the 2,6-di­fluoro­benzoyl and 2,4-di­chloro­benzoyl analogues contain no hydrogen bonds of any sort. Examples of attractive iodo⋯carbonyl inter­actions, as found here in (V), have also been reported in a number of systems (Glidewell *et al.*, 2005 ▸; Garden *et al.*, 2006 ▸; Sirimulla *et al.*, 2013 ▸).

## Synthesis and crystallization   

For the synthesis of compounds (I)–(VII), 1-(3-di­methyl­amino­prop­yl)-3-ethyl­carbodimide (134 mg, 0.7 mmol), 1-hy­droxy­benzotriazole (68 mg, 0.5 mmol) and tri­ethyl­amine (0.5 ml, 1.5 mmol) were added to a solution of the appropriately substituted benzoic acid [benzoic acid for (I), 2-fluoro­benzoic acid for (II), 2-chloro­benzoic acid for (III), 2-bromo­benzoic acid for (IV), 2-iodo­benzoic acid for (V), salicylic acid for (VI) and 4-fluoro­benzoic acid for (VII)] (0.5 mmol) in *N*,*N*-di­methyl­formamide (5 ml) and the resulting mixtures were stirred for 20 min at 273 K. A solution of *N*-(4-meth­oxy­phen­yl)piperazine (100 mg, 0.5 mmol) in *N*,*N*-di­methyl­formamide (5 ml) was then added and stirring was continued overnight at ambient temperature. When the reactions were confirmed to be complete using thin-layer chromatography, each mixture was then quenched with water (10 ml) and extracted with ethyl acetate (20 ml). Each organic fraction was separated and washed successively with an aqueous hydro­chloric acid solution (1 mol dm^−3^), a saturated solution of sodium hydrogencarbonate and then with brine. The organic phases were dried over anhydrous sodium sulfate and the solvent was removed under reduced pressure. Crystals suitable for single-crystal X-ray diffraction were grown by slow evaporation, at ambient temperature and in the presence of air, of solutions in ethyl acetate.

Compound (I). Yield 81%, m.p. 407–409 K. IR (KBr, cm^−1^) 1631 (C=O), 1242 (C—N). NMR (CDCl_3_) δ(^1^H) 3.04 (*t*, 4H, piperazine), 3.56 (*s*, 2H, piperazine), 3.75 (*s*, 3H, O—CH_3_), 3.92 (*s*, 2H, piperazine), 6.83 (*d*, 2H, meth­oxy­phen­yl), 6.89 (*d*, 2H, meth­oxy­phen­yl), 7.42 (*m*, 5H, phen­yl): δ(^13^C) 47.76, 51.22, 55.48 (O—CH_3_), 114.46, 118.88, 127.04, 128.46, 129.72, 135.63, 145.19, 154.36, 170.30.

Compound (II). Yield 80%, m.p. 409–411 K. IR (KBr, cm^−1^) 1626 (C=O), 1242 (C—N). NMR (CDCl_3_) δ(^1^H) 2.99 (*s*, 2H, piprazine), 3.13 (*t*, 2H, piperazine), 3.47 (*s*, 2H, piperazine) 3.75 (*s*, 3H, O—CH_3_), 3.95 (*t*, 2H, piperazine), 6.83 (*d*, 2H, *J* = 9.2 Hz, meth­oxy­phen­yl), 6.89 (*d*, 2H, *J* = 9.2 Hz, meth­oxy­phen­yl), 7.09 (*m*, 1H, 2-fluoro­phen­yl), 7.22 (*m*, 1H, 2-fluoro­phen­yl), 7.40 (*m*, 2H, 2-fluoro­phen­yl): δ(^13^C) 47.08, 51.29, 55.47 (O—CH_3_), 114.47, 118.96, 123.86, 124.60, 129.17, 131.34 145.17, 154.40, 156.82, 159.29, 165.10.

Compound (III). Yield 79%, m.p. 425–427 K. IR (KBr, cm^−1^) 1632 (C=O), 1240 (C—N). NMR (CDCl_3_) δ(^1^H) 2.94 (*m*, 1H, piperazine), 3.07 (*m*, 3H, piperazine), 3.34 (*m*, 1H, piperazine), 3.42 (*m*, 1H, piperazine) 3.75 (*s*, 3H, O—CH_3_), 3.95 (*m*, 2H, piperazine), 6.83 (*d*, 2H, *J* = 9.2Hz, meth­oxy­phen­yl), 6.88 (*t*, 2H, 2-chloro­phen­yl), 7.33 (*m*, 4H, meth­oxy­phenyl and 2-chloro­phen­yl): δ(^13^C) 46.76, 51.22, 55.48 (O—CH_3_), 114.47, 118.94, 127.16, 127.73, 129.63 130.19, 130.31, 135.65, 145.10, 154.41, 166.77.

Compound (IV) Yield 80%, m.p. 410–412 K. IR (KBr, cm^−1^) 1631 (C=O), 1242 (C—N). NMR (CDCl_3_) δ(^1^H) 2.99 (*m*, 1H, piperazine), 3.15 (*m*, 3H, piperazine), 3.38 (*q*, 1H, piperazine), 3.45 (*m*, 1H, piperazine), 3.79 (*s*, 3H, O—CH_3_), 3.99 (*m*, 2H, piperazine), 6.86 (*d*, 2H, *J* = 9.2 Hz, meth­oxy­phen­yl), 6.92 (*d*, 2H, *J* = 9.2 Hz, meth­oxy­phen­yl), 7.29 (*m*, 2H, 2-bromo­phen­yl), 7.39 (*t*, 1H, 2-bromo­phen­yl), 7.61 (*d*, 1H, *J* = 8 Hz, 2-bromo­phen­yl): δ(^13^C) 41.72, 46.86, 50.88, 51.23, 55.54 (O—CH_3_), 114.53, 119.00, 119.19, 127.75, 130.32, 132.85, 137.91, 145.20, 154.47.

Compound (V). Yield 79%, m.p. 423–425 K. IR (KBr, cm^−1^) 1630 (C=O), 1243 (C—N). NMR (CDCl_3_) δ(^1^H) 2.93 (*m*, 1H, piperazine), 3.16 (*m*, 3H, piperazine), 3.32 (*m*, 1H, piperazine), 3.42 (*m*, 1H, piperazine), 3.75 (*s*, 3H, O—CH_3_), 3.96 (*m*, 2H, piperazine), 6.83 (*d*, 2H, *J* = 8.8Hz, meth­oxy­phen­yl), 6.89 (*d*, 2H, *J* = 8.8Hz, meth­oxy­phen­yl), 7.08 (*m*, 1H, 2-iodo­phen­yl), 7.22 (*m*,1H, 2-iodo­phen­yl), 7.39 (*m*, 1H, 2-iodo­phen­yl), 7.83 (*m*, 1H, 2-iodo­phen­yl): δ(^13^C) 46.92, 51.12, 55.49 (O—CH_3_), 92.48, 114.46, 118.95, 127.02, 128.37, 130.24, 139.22, 142.03, 145.12, 154.39

Compound (VI). Yield 79%, m.p. 465–467 K. IR (KBr, cm^−1^) 1631 (C=O), 1228 (C—N). NMR (CDCl_3_) δ(^1^H) 3.10 (*m*, 4H, piperazine), 3.76 (*s*, 3H, O—CH_3_), 3.88 (*m*, 4H, piperazine), 6.85 (*m*, 5H, meth­oxy­phenyl and 2-hy­droxy­phen­yl), 7.01 (*m*, 1H, 2-hy­droxy­phen­yl), 7.26 (*m*,1H, 2-hy­droxy­phen­yl), 7.33 (*m*,1H, 2-hy­droxy­phen­yl): δ(^13^C) 45.84, 51.16, 55.48 (O—CH_3_), 114.51, 116.77, 118.10, 118.53, 118.89, 128.26, 132.69, 145.09, 154.47, 159.09, 170.83.

Compound (VII). Yield 78%, m.p. 401–403 K. IR (KBr, cm^−1^) 1625 (C=O), 1247 (C—N). NMR (CDCl_3_) δ(^1^H) 3.05 (*s*, 4H, piperazine), 3.62 (*m*, 2H, piperazine), 3.75 (*s*, 3H, O—CH_3_), 3.85 (*m*, 2H, piperazine), 6.86 (*m*, 4H, meth­oxy­phen­yl), 7.09 (*m*, 2H, 4-fluoro­phen­yl), 7.44 (*m*, 2H, 4-fluoro­phen­yl): δ(^13^C) 47.77, 51.15, 55.47 (O—CH_3_), 114.47, 115.44, 118.90, 129.43 131.59, 145.11, 154.41, 162.13, 169.39.

## Refinement   

Crystal data, data collection and structure refinement details are summarized in Table 2 ▸. Two bad outlier reflections, (080) and (186), were omitted from the final refinements for compound (V). For the minor disorder component of compound (III), the bonded distances and the 1,3 non-bonded distances were restrained to be the same as those in the major disorder component, subject to s.u. values of 0.01 and 0.02 Å, respectively. The anisotropic displacement parameters for pairs of partial-occupancy atoms occupying essentially the same physical space were constrained to be the same: in addition it was found desirable to constrain the minor component of the chloroaryl ring to be planar, and to apply a rigid-bond restraint to the bond C32—Cl32 in the minor disorder component. Subject to these conditions, the occupancies of the two disorder components refined to 0.942 (2) and 0.058 (2), respectively. After refinement of (IV) as a fully ordered structure, the difference map contained indications of some slight disorder similar to that found for (III). However, when this structure was refined using a disorder model analogous to that used for (III), the preliminary values of the occupancies were 0.9837 (7) and 0.0163 (7), so that each C atom in the minor disorder component represented less than 0.1 electron: accordingly, it was regarded as unrealistic to pursue this disorder model and that the fully ordered model was preferable. The principal feature in the difference map for (V) is a minimum, −2.24 e Å^−3^, located 1.80 Å from atom I2 at (*x*, *y*, *z*) and 1.83 Å from atom O17 at (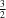 − *x*, 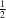 + *y*, *z*), although not co-linear with these two atoms, which subtend an angle of 135° at the minimum. All H atoms apart from those in the minor disorder components of compound (III) were located in difference maps. The H atoms bonded to C atoms were all then treated as riding atoms in geometrically idealized positions with C—H distances of 0.93 Å (aromatic), 0.96 Å (CH_3_) or 0.97 Å (CH_2_), and with *U*_iso_(H) = *kU*_eq_(C), where *k* = 1.5 for the methyl groups, which were permitted to rotate but not to tilt, and 1.2 for all other H atoms bonded to C atoms. For the H atom bonded to an O atom in compound (VI), the atomic coordinates were refined with *U*_iso_(H) = 1.5*U*_eq_(O), giving an O—H distance of 0.92 (2) Å. In the absence of significant resonant scattering in (I), it was not possible to determine the correct orientation of the structure of (I) relative to the polar axis directions: however, this has no chemical significance.

## Supplementary Material

Crystal structure: contains datablock(s) global, I, II, III, IV, V, VI. DOI: 10.1107/S2056989019010491/zl2757sup1.cif

Structure factors: contains datablock(s) I. DOI: 10.1107/S2056989019010491/zl2757Isup2.hkl

Structure factors: contains datablock(s) II. DOI: 10.1107/S2056989019010491/zl2757IIsup3.hkl

Structure factors: contains datablock(s) III. DOI: 10.1107/S2056989019010491/zl2757IIIsup4.hkl

Structure factors: contains datablock(s) IV. DOI: 10.1107/S2056989019010491/zl2757IVsup5.hkl

Structure factors: contains datablock(s) V. DOI: 10.1107/S2056989019010491/zl2757Vsup6.hkl

Structure factors: contains datablock(s) VI. DOI: 10.1107/S2056989019010491/zl2757VIsup7.hkl

Supporting information file. DOI: 10.1107/S2056989019010491/zl2757Isup8.cml

Supporting information file. DOI: 10.1107/S2056989019010491/zl2757IIsup9.cml

Supporting information file. DOI: 10.1107/S2056989019010491/zl2757IIIsup10.cml

Supporting information file. DOI: 10.1107/S2056989019010491/zl2757Vsup11.cml

Supporting information file. DOI: 10.1107/S2056989019010491/zl2757VIsup12.cml

CCDC references: 1942579, 1942578, 1942577, 1942576, 1942575, 1942574

Additional supporting information:  crystallographic information; 3D view; checkCIF report

## Figures and Tables

**Figure 2:**
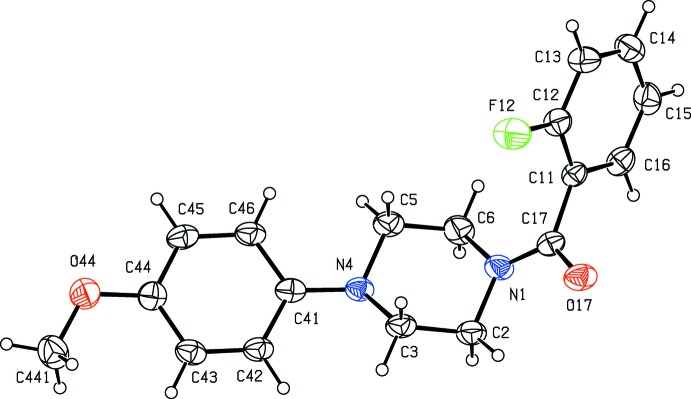
The mol­ecular structure of compound (II) showing the atom-labelling scheme. Displacement ellipsoids are drawn at the 30% probability level.

**Figure 4:**
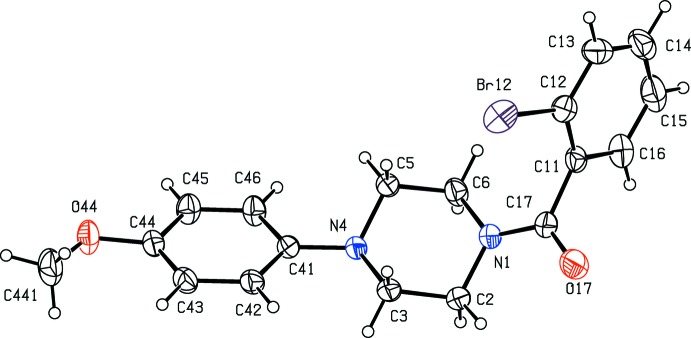
The mol­ecular structure of compound (IV) showing the atom-labelling scheme. Displacement ellipsoids are drawn at the 30% probability level.

**Figure 5:**
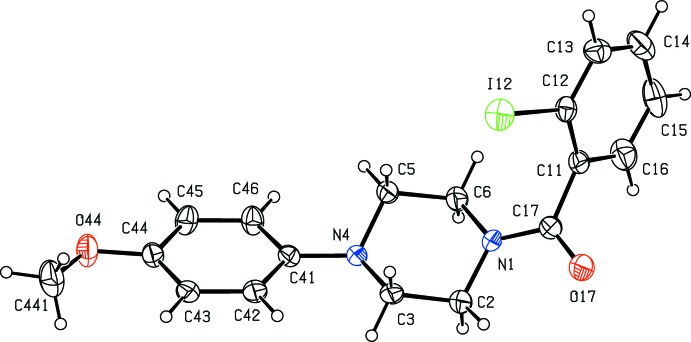
The mol­ecular structure of compound (V) showing the atom-labelling scheme. Displacement ellipsoids are drawn at the 30% probability level.

**Figure 6:**
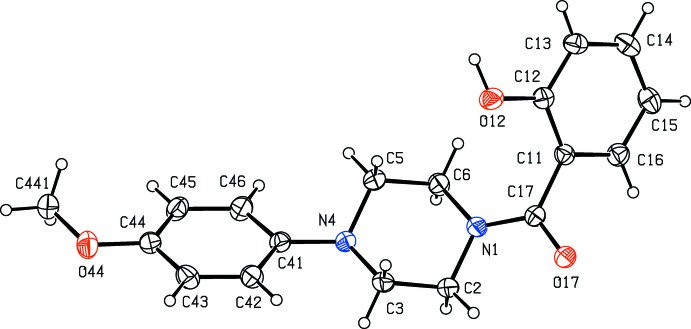
The mol­ecular structure of compound (VI) showing the atom-labelling scheme. Displacement ellipsoids are drawn at the 30% probability level.

**Table 1:** Hydrogen bonds and short inter­molecular contacts (Å, °) in compounds (I)–(VI) *Cg*1 and *Cg*2 are the centroids of the C11–C16 and C41–C46 rings, respectively.

Compound	*D*—H⋯*A*	*D*—H	H⋯*A*	*D*⋯*A*	*D*—H⋯*A*
(I)	C12—H12⋯O17^i^	0.93	2.61	3.497 (4)	160
(II)	C2—H2*A*⋯O17^ii^	0.97	2.50	3.387 (4)	152
	C16—H16⋯O17^iii^	0.93	2.43	3.340 (5)	167
(III)	C3—H3*A*⋯O17^iv^	0.97	2.61	3.574 (3)	175
	C2—HA⋯*Cg*1^iv^	0.97	2.84	3.648 (3)	142
	C15—H15⋯*Cg*2^v^	0.93	2.72	3.610 (4)	162
(IV)	C3—H3*A*⋯O17^iv^	0.97	2.56	3.524 (3)	171
	C2—HA⋯*Cg*1^iv^	0.97	2.82	3.630 (3)	142
	C15—H15⋯*Cg*2^v^	0.93	2.68	3.579 (4)	164
(V)	C3—H3*A*⋯O17^iv^	0.97	2.60	3.542 (10)	164
	C2—H*A*⋯*Cg*1^iv^	0.97	2.87	3.719 (11)	147
	C15—H15⋯*Cg*2^v^	0.93	2.73	3.656 (12)	172
(VI)	O12—H12⋯O17^vi^	0.92 (2)	1.81 (2)	3.7327 (15)	175.4 (18)

Symmetry codes: (i) *x*, 1 − *y*, −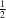 + *z*; (ii) 1 − *x*, 1 − *y*, 1 − *z*; (iii) 2 − *x*, 1 − *y*, 1 − *z*; (iv) −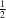 + *x*, 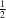 − *y*, 1 − *z*; (v) 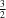 − *x*, −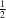 + *y*, *z*; (vi) −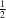 + *x*, *y*, 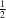 − *z*.

**Table:** 

	(I)	(II)	(III)
Crystal data			
Chemical formula	C_18_H_20_N_2_O_2_	C_18_H_19_FN_2_O_2_	C_18_H_19_ClN_2_O_2_
*M*_r_	296.36	314.35	330.80
Crystal system, space group	Monoclinic, *C* *c*	Monoclinic, *P*2_1_/*c*	Orthorhombic, *P* *b* *c* *a*
Temperature (K)	293	293	293
*a*, *b*, *c* (Å)	29.403 (5), 7.9811 (14), 6.7898 (13)	6.998 (2), 7.938 (2), 28.415 (6)	13.0320 (11), 13.2470 (13), 19.258 (2)
α, β, γ (°)	90, 97.352 (12), 90	90, 92.20 (3), 90	90, 90, 90
*V* (Å^3^)	1580.3 (5)	1577.3 (7)	3324.6 (6)
*Z*	4	4	8
Radiation type	Mo *K*α	Mo *K*α	Mo *K*α
μ (mm^−1^)	0.08	0.10	0.24
Crystal size (mm)	0.48 × 0.48 × 0.28	0.48 × 0.36 × 0.32	0.50 × 0.40 × 0.38

Data collection			
Diffractometer	Oxford Diffraction Xcalibur diffractometer with Sapphire CCD	Oxford Diffraction Xcalibur diffractometer with Sapphire CCD	Oxford Diffraction Xcalibur diffractometer with Sapphire CCD
Absorption correction	Multi-scan (*CrysAlis RED*; Oxford Diffraction, 2009 ▸)	Multi-scan (*CrysAlis RED*; Oxford Diffraction, 2009 ▸)	Multi-scan (*CrysAlis RED*; Oxford Diffraction, 2009 ▸)
*T*_min_, *T*_max_	0.951, 0.977	0.931, 0.970	0.862, 0.912
No. of measured, independent and observed [*I* > 2σ(*I*)] reflections	5476, 2137, 1766	6039, 3315, 1863	13862, 3642, 2407
*R*_int_	0.019	0.049	0.022
(sin θ/λ)_max_ (Å^−1^)	0.655	0.651	0.656

Refinement			
*R*[*F* ^2^ > 2σ(*F* ^2^)], *wR*(*F* ^2^), *S*	0.035, 0.089, 1.08	0.070, 0.190, 1.08	0.048, 0.127, 1.03
No. of reflections	2137	3315	3642
No. of parameters	201	208	243
No. of restraints	2	0	26
H-atom treatment	H-atom parameters constrained	H-atom parameters constrained	H-atom parameters constrained
Δρ_max_, Δρ_min_ (e Å^−3^)	0.14, −0.13	0.21, −0.27	0.23, −0.45

**Table:** 

	(IV)	(V)	(VI)
Crystal data			
Chemical formula	C_18_H_19_BrN_2_O_2_	C_18_H_19_IN_2_O_2_	C_18_H_20_N_2_O_3_
*M*_r_	375.26	422.25	312.36
Crystal system, space group	Orthorhombic, *P* *b* *c* *a*	Orthorhombic, *P* *b* *c* *a*	Orthorhombic, *P* *b* *c* *a*
Temperature (K)	293	293	293
*a*, *b*, *c* (Å)	12.9119 (14), 13.3664 (16), 19.5019 (19)	12.7671 (13), 13.5429 (12), 20.2542 (16)	9.7265 (6), 12.9084 (9), 24.861 (1)
α, β, γ (°)	90, 90, 90	90, 90, 90	90, 90, 90
*V* (Å^3^)	3365.7 (6)	3502.0 (5)	3121.4 (3)
*Z*	8	8	8
Radiation type	Mo *K*α	Mo *K*α	Mo *K*α
μ (mm^−1^)	2.45	1.84	0.09
Crystal size (mm)	0.22 × 0.21 × 0.18	0.48 × 0.42 × 0.38	0.50 × 0.40 × 0.16

Data collection			
Diffractometer	Bruker D8 Quest	Oxford Diffraction Xcalibur diffractometer with Sapphire CCD	Oxford Diffraction Xcalibur diffractometer with Sapphire CCD
Absorption correction	Multi-scan (*SADABS*; Bruker, 2015 ▸	Multi-scan (*CrysAlis RED*; Oxford Diffraction, 2009 ▸)	Multi-scan (*CrysAlis RED*; Oxford Diffraction, 2009 ▸)
*T*_min_, *T*_max_	0.538, 0.643	0.408, 0.497	0.917, 0.986
No. of measured, independent and observed [*I* > 2σ(*I*)] reflections	47663, 4262, 3135	14215, 3838, 3062	11981, 3474, 2492
*R*_int_	0.039	0.029	0.020
(sin θ/λ)_max_ (Å^−1^)	0.672	0.655	0.658

Refinement			
*R*[*F* ^2^ > 2σ(*F* ^2^)], *wR*(*F* ^2^), *S*	0.045, 0.131, 1.02	0.068, 0.146, 1.18	0.041, 0.100, 1.04
No. of reflections	4262	3838	3474
No. of parameters	209	209	212
No. of restraints	0	0	0
H-atom treatment	H-atom parameters constrained	H-atom parameters constrained	H atoms treated by a mixture of independent and constrained refinement
Δρ_max_, Δρ_min_ (e Å^−3^)	0.54, −0.64	1.27, −2.19	0.16, −0.17

Computer programs: *CrysAlis CCD* and *CrysAlis RED* (Oxford Diffraction, 2009 ▸), *APEX2* and *SAINT* (Bruker, 2015 ▸), *SHELXT* (Sheldrick, 2015*a* ▸), *SHELXL2014* (Sheldrick, 2015*b* ▸) and *PLATON* (Spek, 2009 ▸).

## supplementary crystallographic information

### 1-Benzoyl-4-(4-methoxyphenyl)piperazine (I) Crystal data

**Table:**

C_18_H_20_N_2_O_2_	*F*(000) = 632
*M**_r_* = 296.36	*D*_x_ = 1.246 Mg m^−^^3^
Monoclinic, *C**c*	Mo *K*α radiation, λ = 0.71073 Å
*a* = 29.403 (5) Å	Cell parameters from 2137 reflections
*b* = 7.9811 (14) Å	θ = 2.7–27.7°
*c* = 6.7898 (13) Å	µ = 0.08 mm^−^^1^
β = 97.352 (12)°	*T* = 293 K
*V* = 1580.3 (5) Å^3^	Plate, colourless
*Z* = 4	0.48 × 0.48 × 0.28 mm

### 1-Benzoyl-4-(4-methoxyphenyl)piperazine (I) Data collection

**Table:**

Oxford Diffraction Xcalibur diffractometer with Sapphire CCD	2137 independent reflections
Radiation source: Enhance (Mo) X-ray Source	1766 reflections with *I* > 2σ(*I*)
Graphite monochromator	*R*_int_ = 0.019
ω scans	θ_max_ = 27.7°, θ_min_ = 2.7°
Absorption correction: multi-scan (CrysAlis RED; Oxford Diffraction, 2009)	*h* = −37→37
*T*_min_ = 0.951, *T*_max_ = 0.977	*k* = −10→10
5476 measured reflections	*l* = −4→8

### 1-Benzoyl-4-(4-methoxyphenyl)piperazine (I) Refinement

**Table:**

Refinement on *F*^2^	Hydrogen site location: inferred from neighbouring sites
Least-squares matrix: full	H-atom parameters constrained
*R*[*F*^2^ > 2σ(*F*^2^)] = 0.035	*w* = 1/[σ^2^(*F*_o_^2^) + (0.0414*P*)^2^ + 0.3311*P*] where *P* = (*F*_o_^2^ + 2*F*_c_^2^)/3
*wR*(*F*^2^) = 0.089	(Δ/σ)_max_ < 0.001
*S* = 1.08	Δρ_max_ = 0.14 e Å^−^^3^
2137 reflections	Δρ_min_ = −0.13 e Å^−^^3^
201 parameters	Extinction correction: SHELXL, Fc^*^=kFc[1+0.001xFc^2^λ^3^/sin(2θ)]^-1/4^
2 restraints	Extinction coefficient: 0.0041 (8)
Primary atom site location: difference Fourier map	

### 1-Benzoyl-4-(4-methoxyphenyl)piperazine (I) Special details

**Table:**

Geometry. All esds (except the esd in the dihedral angle between two l.s. planes) are estimated using the full covariance matrix. The cell esds are taken into account individually in the estimation of esds in distances, angles and torsion angles; correlations between esds in cell parameters are only used when they are defined by crystal symmetry. An approximate (isotropic) treatment of cell esds is used for estimating esds involving l.s. planes.

### 1-Benzoyl-4-(4-methoxyphenyl)piperazine (I) Fractional atomic coordinates and isotropic or equivalent isotropic displacement parameters (Å^2^)

**Table:**

	*x*	*y*	*z*	*U*_iso_*/*U*_eq_	
N1	0.46344 (7)	0.2520 (2)	0.4700 (3)	0.0433 (5)	
C2	0.41820 (8)	0.2497 (3)	0.5365 (3)	0.0439 (6)	
H2A	0.4189	0.3164	0.6563	0.053*	
H2B	0.4105	0.1356	0.5686	0.053*	
C3	0.38166 (9)	0.3177 (3)	0.3801 (3)	0.0440 (6)	
H3A	0.3518	0.3068	0.4254	0.053*	
H3B	0.3872	0.4358	0.3589	0.053*	
N4	0.38189 (7)	0.2271 (2)	0.1945 (3)	0.0391 (5)	
C5	0.42693 (8)	0.2452 (3)	0.1240 (3)	0.0437 (6)	
H5A	0.4329	0.3627	0.1012	0.052*	
H5B	0.4268	0.1862	−0.0009	0.052*	
C6	0.46440 (9)	0.1755 (3)	0.2747 (4)	0.0453 (6)	
H6A	0.4606	0.0552	0.2850	0.054*	
H6B	0.4940	0.1963	0.2304	0.054*	
C17	0.50023 (9)	0.2585 (3)	0.6128 (4)	0.0458 (6)	
O17	0.49611 (7)	0.2708 (3)	0.7894 (3)	0.0697 (6)	
C11	0.54739 (9)	0.2548 (3)	0.5486 (4)	0.0479 (6)	
C12	0.56066 (10)	0.3679 (4)	0.4144 (5)	0.0638 (8)	
H12	0.5399	0.4469	0.3558	0.077*	
C13	0.60510 (11)	0.3637 (5)	0.3668 (6)	0.0780 (10)	
H13	0.6143	0.4415	0.2778	0.094*	
C14	0.63520 (11)	0.2479 (5)	0.4481 (5)	0.0754 (10)	
H14	0.6646	0.2443	0.4120	0.091*	
C15	0.62257 (11)	0.1350 (5)	0.5841 (5)	0.0776 (10)	
H15	0.6435	0.0561	0.6411	0.093*	
C16	0.57874 (10)	0.1391 (4)	0.6357 (4)	0.0626 (8)	
H16	0.5702	0.0641	0.7292	0.075*	
C41	0.34279 (8)	0.2454 (3)	0.0494 (3)	0.0382 (6)	
C42	0.30660 (8)	0.3523 (3)	0.0761 (4)	0.0454 (6)	
H42	0.3089	0.4215	0.1870	0.055*	
C43	0.26735 (9)	0.3569 (3)	−0.0600 (4)	0.0497 (7)	
H43	0.2435	0.4282	−0.0379	0.060*	
C44	0.26289 (8)	0.2581 (4)	−0.2277 (4)	0.0474 (6)	
C45	0.29861 (9)	0.1527 (3)	−0.2590 (4)	0.0464 (6)	
H45	0.2963	0.0857	−0.3719	0.056*	
C46	0.33796 (9)	0.1470 (3)	−0.1215 (4)	0.0444 (6)	
H46	0.3617	0.0754	−0.1443	0.053*	
O44	0.22252 (7)	0.2728 (3)	−0.3541 (3)	0.0671 (6)	
C441	0.21605 (15)	0.1672 (6)	−0.5223 (6)	0.0907 (12)	
H41A	0.1855	0.1811	−0.5889	0.136*	
H41B	0.2206	0.0527	−0.4811	0.136*	
H41C	0.2377	0.1961	−0.6112	0.136*	

### 1-Benzoyl-4-(4-methoxyphenyl)piperazine (I) Atomic displacement parameters (Å^2^)

**Table:**

	*U*^11^	*U*^22^	*U*^33^	*U*^12^	*U*^13^	*U*^23^
N1	0.0391 (11)	0.0513 (13)	0.0412 (11)	−0.0030 (10)	0.0121 (9)	−0.0049 (9)
C2	0.0445 (14)	0.0484 (14)	0.0410 (14)	−0.0017 (12)	0.0139 (11)	−0.0028 (11)
C3	0.0435 (13)	0.0484 (14)	0.0429 (14)	0.0032 (12)	0.0163 (11)	−0.0018 (11)
N4	0.0364 (11)	0.0447 (11)	0.0384 (11)	0.0040 (10)	0.0136 (9)	−0.0015 (9)
C5	0.0447 (14)	0.0510 (15)	0.0380 (13)	0.0017 (11)	0.0154 (11)	0.0001 (10)
C6	0.0413 (13)	0.0521 (14)	0.0442 (13)	0.0038 (12)	0.0124 (11)	−0.0035 (11)
C17	0.0454 (15)	0.0482 (15)	0.0448 (15)	−0.0057 (13)	0.0093 (12)	−0.0004 (11)
O17	0.0558 (11)	0.1098 (18)	0.0448 (11)	−0.0141 (12)	0.0117 (9)	−0.0072 (11)
C11	0.0444 (14)	0.0553 (16)	0.0440 (14)	−0.0069 (13)	0.0055 (11)	−0.0037 (12)
C12	0.0483 (16)	0.074 (2)	0.0703 (19)	−0.0011 (15)	0.0131 (14)	0.0196 (15)
C13	0.056 (2)	0.103 (3)	0.078 (2)	−0.0136 (18)	0.0197 (17)	0.020 (2)
C14	0.0406 (17)	0.110 (3)	0.078 (2)	−0.0040 (18)	0.0141 (16)	−0.005 (2)
C15	0.0518 (19)	0.093 (3)	0.085 (2)	0.0120 (18)	−0.0028 (16)	0.0019 (19)
C16	0.0510 (17)	0.074 (2)	0.0608 (18)	−0.0008 (15)	0.0009 (14)	0.0100 (15)
C41	0.0386 (13)	0.0376 (13)	0.0407 (13)	−0.0028 (11)	0.0144 (11)	0.0050 (10)
C42	0.0402 (14)	0.0471 (16)	0.0511 (15)	0.0014 (11)	0.0143 (11)	−0.0046 (11)
C43	0.0396 (14)	0.0489 (16)	0.0633 (17)	0.0069 (13)	0.0165 (13)	0.0004 (13)
C44	0.0371 (14)	0.0555 (16)	0.0504 (15)	−0.0032 (13)	0.0093 (11)	0.0081 (13)
C45	0.0504 (15)	0.0488 (16)	0.0413 (14)	−0.0014 (13)	0.0117 (11)	−0.0033 (11)
C46	0.0468 (14)	0.0449 (15)	0.0439 (14)	0.0085 (12)	0.0155 (11)	0.0000 (11)
O44	0.0461 (11)	0.0883 (16)	0.0651 (13)	0.0030 (11)	0.0007 (9)	−0.0011 (12)
C441	0.076 (2)	0.110 (3)	0.078 (3)	0.001 (2)	−0.0201 (18)	−0.015 (2)

### 1-Benzoyl-4-(4-methoxyphenyl)piperazine (I) Geometric parameters (Å, º)

**Table:**

N1—C17	1.358 (3)	C13—C14	1.348 (5)
N1—C2	1.458 (3)	C13—H13	0.9300
N1—C6	1.463 (3)	C14—C15	1.375 (5)
C2—C3	1.511 (4)	C14—H14	0.9300
C2—H2A	0.9700	C15—C16	1.378 (4)
C2—H2B	0.9700	C15—H15	0.9300
C3—N4	1.454 (3)	C16—H16	0.9300
C3—H3A	0.9700	C41—C46	1.393 (4)
C3—H3B	0.9700	C41—C42	1.394 (3)
N4—C41	1.422 (3)	C42—C43	1.384 (4)
N4—C5	1.472 (3)	C42—H42	0.9300
C5—C6	1.511 (4)	C43—C44	1.378 (4)
C5—H5A	0.9700	C43—H43	0.9300
C5—H5B	0.9700	C44—O44	1.378 (3)
C6—H6A	0.9700	C44—C45	1.383 (4)
C6—H6B	0.9700	C45—C46	1.391 (4)
C17—O17	1.225 (3)	C45—H45	0.9300
C17—C11	1.506 (3)	C46—H46	0.9300
C11—C12	1.374 (4)	O44—C441	1.412 (4)
C11—C16	1.383 (4)	C441—H41A	0.9600
C12—C13	1.386 (4)	C441—H41B	0.9600
C12—H12	0.9300	C441—H41C	0.9600
			
C17—N1—C2	117.04 (19)	C13—C12—H12	120.2
C17—N1—C6	123.82 (19)	C14—C13—C12	120.7 (3)
C2—N1—C6	113.5 (2)	C14—C13—H13	119.7
N1—C2—C3	111.88 (19)	C12—C13—H13	119.7
N1—C2—H2A	109.2	C13—C14—C15	120.3 (3)
C3—C2—H2A	109.2	C13—C14—H14	119.8
N1—C2—H2B	109.2	C15—C14—H14	119.8
C3—C2—H2B	109.2	C14—C15—C16	119.8 (3)
H2A—C2—H2B	107.9	C14—C15—H15	120.1
N4—C3—C2	110.4 (2)	C16—C15—H15	120.1
N4—C3—H3A	109.6	C15—C16—C11	120.1 (3)
C2—C3—H3A	109.6	C15—C16—H16	120.0
N4—C3—H3B	109.6	C11—C16—H16	120.0
C2—C3—H3B	109.6	C46—C41—C42	117.1 (2)
H3A—C3—H3B	108.1	C46—C41—N4	120.4 (2)
C41—N4—C3	117.17 (18)	C42—C41—N4	122.4 (2)
C41—N4—C5	116.48 (17)	C43—C42—C41	121.0 (2)
C3—N4—C5	109.71 (19)	C43—C42—H42	119.5
N4—C5—C6	110.63 (18)	C41—C42—H42	119.5
N4—C5—H5A	109.5	C44—C43—C42	121.3 (2)
C6—C5—H5A	109.5	C44—C43—H43	119.3
N4—C5—H5B	109.5	C42—C43—H43	119.3
C6—C5—H5B	109.5	C43—C44—O44	116.7 (2)
H5A—C5—H5B	108.1	C43—C44—C45	118.8 (2)
N1—C6—C5	111.3 (2)	O44—C44—C45	124.5 (3)
N1—C6—H6A	109.4	C44—C45—C46	120.0 (2)
C5—C6—H6A	109.4	C44—C45—H45	120.0
N1—C6—H6B	109.4	C46—C45—H45	120.0
C5—C6—H6B	109.4	C45—C46—C41	121.9 (2)
H6A—C6—H6B	108.0	C45—C46—H46	119.1
O17—C17—N1	122.2 (2)	C41—C46—H46	119.1
O17—C17—C11	119.7 (2)	C44—O44—C441	118.0 (3)
N1—C17—C11	118.1 (2)	O44—C441—H41A	109.5
C12—C11—C16	119.5 (3)	O44—C441—H41B	109.5
C12—C11—C17	122.0 (3)	H41A—C441—H41B	109.5
C16—C11—C17	118.5 (2)	O44—C441—H41C	109.5
C11—C12—C13	119.6 (3)	H41A—C441—H41C	109.5
C11—C12—H12	120.2	H41B—C441—H41C	109.5
			
C17—N1—C2—C3	154.8 (2)	C12—C13—C14—C15	−1.9 (6)
C6—N1—C2—C3	−50.5 (3)	C13—C14—C15—C16	0.8 (6)
N1—C2—C3—N4	55.1 (3)	C14—C15—C16—C11	1.0 (5)
C2—C3—N4—C41	164.3 (2)	C12—C11—C16—C15	−1.6 (4)
C2—C3—N4—C5	−59.9 (3)	C17—C11—C16—C15	−178.4 (3)
C41—N4—C5—C6	−163.8 (2)	C3—N4—C41—C46	−172.1 (2)
C3—N4—C5—C6	60.1 (3)	C5—N4—C41—C46	55.1 (3)
C17—N1—C6—C5	−157.0 (2)	C3—N4—C41—C42	3.6 (3)
C2—N1—C6—C5	50.3 (3)	C5—N4—C41—C42	−129.2 (2)
N4—C5—C6—N1	−54.7 (3)	C46—C41—C42—C43	1.3 (4)
C2—N1—C17—O17	−3.7 (4)	N4—C41—C42—C43	−174.5 (2)
C6—N1—C17—O17	−155.6 (3)	C41—C42—C43—C44	−0.9 (4)
C2—N1—C17—C11	177.9 (2)	C42—C43—C44—O44	−179.7 (2)
C6—N1—C17—C11	26.1 (3)	C42—C43—C44—C45	0.0 (4)
O17—C17—C11—C12	−123.5 (3)	C43—C44—C45—C46	0.5 (4)
N1—C17—C11—C12	54.9 (4)	O44—C44—C45—C46	−179.8 (2)
O17—C17—C11—C16	53.2 (4)	C44—C45—C46—C41	−0.1 (4)
N1—C17—C11—C16	−128.4 (3)	C42—C41—C46—C45	−0.8 (4)
C16—C11—C12—C13	0.6 (5)	N4—C41—C46—C45	175.1 (2)
C17—C11—C12—C13	177.2 (3)	C43—C44—O44—C441	−177.0 (3)
C11—C12—C13—C14	1.2 (6)	C45—C44—O44—C441	3.3 (4)

### 1-Benzoyl-4-(4-methoxyphenyl)piperazine (I) Hydrogen-bond geometry (Å, º)

**Table:**

*D*—H···*A*	*D*—H	H···*A*	*D*···*A*	*D*—H···*A*
C12—H12···O17^i^	0.93	2.61	3.497 (4)	160

Symmetry code: (i) *x*, −*y*+1, *z*−1/2.

### 1-(2-Fluorobenzoyl)-4-(4-methoxyphenyl)piperazine (II) Crystal data

**Table:**

C_18_H_19_FN_2_O_2_	*F*(000) = 664
*M**_r_* = 314.35	*D*_x_ = 1.324 Mg m^−^^3^
Monoclinic, *P*2_1_/*c*	Mo *K*α radiation, λ = 0.71073 Å
*a* = 6.998 (2) Å	Cell parameters from 3334 reflections
*b* = 7.938 (2) Å	θ = 2.7–28.4°
*c* = 28.415 (6) Å	µ = 0.10 mm^−^^1^
β = 92.20 (3)°	*T* = 293 K
*V* = 1577.3 (7) Å^3^	Block, colourless
*Z* = 4	0.48 × 0.36 × 0.32 mm

### 1-(2-Fluorobenzoyl)-4-(4-methoxyphenyl)piperazine (II) Data collection

**Table:**

Oxford Diffraction Xcalibur diffractometer with Sapphire CCD	3315 independent reflections
Radiation source: Enhance (Mo) X-ray Source	1863 reflections with *I* > 2σ(*I*)
Graphite monochromator	*R*_int_ = 0.049
ω scans	θ_max_ = 27.6°, θ_min_ = 2.7°
Absorption correction: multi-scan (CrysAlis RED; Oxford Diffraction, 2009)	*h* = −8→5
*T*_min_ = 0.931, *T*_max_ = 0.970	*k* = −10→5
6039 measured reflections	*l* = −36→28

### 1-(2-Fluorobenzoyl)-4-(4-methoxyphenyl)piperazine (II) Refinement

**Table:**

Refinement on *F*^2^	Primary atom site location: difference Fourier map
Least-squares matrix: full	Hydrogen site location: inferred from neighbouring sites
*R*[*F*^2^ > 2σ(*F*^2^)] = 0.070	H-atom parameters constrained
*wR*(*F*^2^) = 0.190	*w* = 1/[σ^2^(*F*_o_^2^) + (0.0566*P*)^2^ + 1.2906*P*] where *P* = (*F*_o_^2^ + 2*F*_c_^2^)/3
*S* = 1.08	(Δ/σ)_max_ < 0.001
3315 reflections	Δρ_max_ = 0.21 e Å^−^^3^
208 parameters	Δρ_min_ = −0.27 e Å^−^^3^
0 restraints	

### 1-(2-Fluorobenzoyl)-4-(4-methoxyphenyl)piperazine (II) Special details

**Table:**

Geometry. All esds (except the esd in the dihedral angle between two l.s. planes) are estimated using the full covariance matrix. The cell esds are taken into account individually in the estimation of esds in distances, angles and torsion angles; correlations between esds in cell parameters are only used when they are defined by crystal symmetry. An approximate (isotropic) treatment of cell esds is used for estimating esds involving l.s. planes.

### 1-(2-Fluorobenzoyl)-4-(4-methoxyphenyl)piperazine (II) Fractional atomic coordinates and isotropic or equivalent isotropic displacement parameters (Å^2^)

**Table:**

	*x*	*y*	*z*	*U*_iso_*/*U*_eq_	
N1	0.7575 (4)	0.5325 (4)	0.41972 (9)	0.0547 (8)	
C2	0.5981 (5)	0.4154 (5)	0.42224 (11)	0.0560 (9)	
H2A	0.5401	0.4256	0.4526	0.067*	
H2B	0.6439	0.3008	0.4191	0.067*	
C3	0.4517 (5)	0.4532 (5)	0.38354 (11)	0.0541 (9)	
H3A	0.3490	0.3714	0.3845	0.065*	
H3B	0.3976	0.5639	0.3887	0.065*	
N4	0.5348 (3)	0.4485 (3)	0.33706 (9)	0.0475 (7)	
C5	0.6957 (5)	0.5657 (5)	0.33544 (11)	0.0567 (9)	
H5A	0.6492	0.6801	0.3386	0.068*	
H5B	0.7543	0.5563	0.3052	0.068*	
C6	0.8428 (5)	0.5301 (5)	0.37396 (11)	0.0623 (10)	
H6A	0.8999	0.4207	0.3688	0.075*	
H6B	0.9433	0.6142	0.3733	0.075*	
C17	0.7990 (4)	0.6422 (4)	0.45441 (11)	0.0464 (8)	
O17	0.7152 (3)	0.6409 (3)	0.49118 (8)	0.0632 (7)	
C11	0.9578 (4)	0.7658 (4)	0.44823 (10)	0.0436 (8)	
C12	0.9203 (4)	0.9299 (5)	0.43559 (11)	0.0516 (8)	
F12	0.7371 (3)	0.9715 (3)	0.42311 (8)	0.0805 (7)	
C13	1.0575 (5)	1.0534 (5)	0.43537 (12)	0.0628 (10)	
H13	1.0261	1.1635	0.4270	0.075*	
C14	1.2416 (5)	1.0101 (5)	0.44775 (12)	0.0630 (10)	
H14	1.3371	1.0916	0.4479	0.076*	
C15	1.2868 (5)	0.8473 (6)	0.45996 (12)	0.0623 (10)	
H15	1.4129	0.8190	0.4678	0.075*	
C16	1.1467 (5)	0.7253 (5)	0.46063 (11)	0.0542 (9)	
H16	1.1785	0.6156	0.4694	0.065*	
C41	0.4022 (4)	0.4532 (4)	0.29834 (11)	0.0466 (8)	
C42	0.2375 (5)	0.3540 (4)	0.29751 (11)	0.0516 (8)	
H42	0.2144	0.2861	0.3234	0.062*	
C43	0.1076 (5)	0.3529 (5)	0.25972 (11)	0.0545 (9)	
H43	−0.0010	0.2857	0.2606	0.065*	
C44	0.1383 (5)	0.4511 (5)	0.22074 (11)	0.0529 (9)	
C45	0.3014 (5)	0.5483 (5)	0.22029 (12)	0.0567 (9)	
H45	0.3256	0.6126	0.1938	0.068*	
C46	0.4291 (5)	0.5518 (4)	0.25840 (11)	0.0538 (9)	
H46	0.5358	0.6214	0.2575	0.065*	
O44	0.0183 (4)	0.4587 (4)	0.18145 (8)	0.0718 (8)	
C441	−0.1580 (5)	0.3703 (6)	0.18248 (13)	0.0756 (12)	
H41A	−0.2281	0.3857	0.1531	0.113*	
H41B	−0.1334	0.2525	0.1874	0.113*	
H41C	−0.2317	0.4128	0.2077	0.113*	

### 1-(2-Fluorobenzoyl)-4-(4-methoxyphenyl)piperazine (II) Atomic displacement parameters (Å^2^)

**Table:**

	*U*^11^	*U*^22^	*U*^33^	*U*^12^	*U*^13^	*U*^23^
N1	0.0531 (16)	0.062 (2)	0.0495 (15)	−0.0161 (15)	0.0145 (12)	−0.0028 (14)
C2	0.059 (2)	0.054 (2)	0.0556 (19)	−0.0119 (18)	0.0113 (16)	0.0044 (17)
C3	0.0531 (19)	0.058 (2)	0.0528 (19)	−0.0110 (18)	0.0184 (15)	0.0043 (16)
N4	0.0481 (14)	0.0478 (18)	0.0474 (14)	−0.0074 (13)	0.0150 (12)	−0.0020 (13)
C5	0.0568 (19)	0.065 (2)	0.0496 (18)	−0.0130 (19)	0.0224 (16)	−0.0017 (17)
C6	0.0518 (19)	0.081 (3)	0.056 (2)	−0.013 (2)	0.0173 (16)	−0.0064 (19)
C17	0.0458 (17)	0.046 (2)	0.0478 (18)	0.0034 (16)	0.0095 (14)	0.0067 (16)
O17	0.0736 (16)	0.0618 (17)	0.0561 (13)	−0.0101 (13)	0.0245 (12)	−0.0006 (12)
C11	0.0442 (17)	0.048 (2)	0.0396 (15)	0.0010 (15)	0.0066 (12)	0.0047 (14)
C12	0.0423 (17)	0.055 (2)	0.058 (2)	0.0062 (17)	0.0018 (14)	0.0089 (17)
F12	0.0519 (12)	0.0683 (16)	0.1207 (19)	0.0103 (11)	−0.0041 (11)	0.0252 (13)
C13	0.065 (2)	0.049 (2)	0.074 (2)	−0.003 (2)	0.0074 (18)	0.0086 (19)
C14	0.060 (2)	0.069 (3)	0.061 (2)	−0.017 (2)	0.0079 (17)	−0.009 (2)
C15	0.0420 (18)	0.089 (3)	0.056 (2)	−0.001 (2)	0.0027 (15)	−0.001 (2)
C16	0.0522 (19)	0.058 (2)	0.0523 (19)	0.0106 (18)	0.0058 (15)	0.0086 (17)
C41	0.0496 (18)	0.0407 (19)	0.0511 (18)	−0.0006 (16)	0.0218 (14)	−0.0056 (15)
C42	0.061 (2)	0.047 (2)	0.0485 (18)	−0.0089 (18)	0.0179 (15)	−0.0018 (16)
C43	0.0552 (19)	0.051 (2)	0.058 (2)	−0.0096 (18)	0.0163 (16)	−0.0074 (18)
C44	0.0569 (19)	0.055 (2)	0.0479 (18)	0.0019 (18)	0.0114 (16)	−0.0065 (17)
C45	0.064 (2)	0.053 (2)	0.054 (2)	−0.0051 (19)	0.0201 (17)	0.0065 (17)
C46	0.0523 (18)	0.053 (2)	0.057 (2)	−0.0109 (18)	0.0163 (16)	0.0021 (17)
O44	0.0685 (16)	0.087 (2)	0.0598 (15)	−0.0122 (15)	0.0054 (12)	0.0035 (14)
C441	0.063 (2)	0.097 (3)	0.067 (2)	−0.014 (2)	0.0057 (18)	−0.009 (2)

### 1-(2-Fluorobenzoyl)-4-(4-methoxyphenyl)piperazine (II) Geometric parameters (Å, º)

**Table:**

N1—C17	1.338 (4)	C13—C14	1.367 (5)
N1—C6	1.452 (4)	C13—H13	0.9300
N1—C2	1.456 (4)	C14—C15	1.371 (5)
C2—C3	1.504 (4)	C14—H14	0.9300
C2—H2A	0.9700	C15—C16	1.379 (5)
C2—H2B	0.9700	C15—H15	0.9300
C3—N4	1.464 (4)	C16—H16	0.9300
C3—H3A	0.9700	C41—C42	1.396 (4)
C3—H3B	0.9700	C41—C46	1.397 (4)
N4—C41	1.412 (4)	C42—C43	1.380 (4)
N4—C5	1.462 (4)	C42—H42	0.9300
C5—C6	1.500 (5)	C43—C44	1.378 (4)
C5—H5A	0.9700	C43—H43	0.9300
C5—H5B	0.9700	C44—O44	1.373 (4)
C6—H6A	0.9700	C44—C45	1.378 (5)
C6—H6B	0.9700	C45—C46	1.378 (5)
C17—O17	1.218 (3)	C45—H45	0.9300
C17—C11	1.498 (4)	C46—H46	0.9300
C11—C12	1.374 (5)	O44—C441	1.421 (4)
C11—C16	1.393 (4)	C441—H41A	0.9600
C12—F12	1.358 (4)	C441—H41B	0.9600
C12—C13	1.372 (5)	C441—H41C	0.9600
			
C17—N1—C6	125.6 (3)	C13—C12—C11	123.5 (3)
C17—N1—C2	121.6 (3)	C14—C13—C12	118.2 (4)
C6—N1—C2	112.2 (3)	C14—C13—H13	120.9
N1—C2—C3	109.8 (3)	C12—C13—H13	120.9
N1—C2—H2A	109.7	C13—C14—C15	120.5 (4)
C3—C2—H2A	109.7	C13—C14—H14	119.7
N1—C2—H2B	109.7	C15—C14—H14	119.7
C3—C2—H2B	109.7	C14—C15—C16	120.6 (3)
H2A—C2—H2B	108.2	C14—C15—H15	119.7
N4—C3—C2	111.8 (3)	C16—C15—H15	119.7
N4—C3—H3A	109.2	C15—C16—C11	120.2 (3)
C2—C3—H3A	109.2	C15—C16—H16	119.9
N4—C3—H3B	109.2	C11—C16—H16	119.9
C2—C3—H3B	109.2	C42—C41—C46	116.1 (3)
H3A—C3—H3B	107.9	C42—C41—N4	121.0 (3)
C41—N4—C5	116.3 (2)	C46—C41—N4	122.8 (3)
C41—N4—C3	115.5 (2)	C43—C42—C41	122.4 (3)
C5—N4—C3	110.2 (2)	C43—C42—H42	118.8
N4—C5—C6	111.4 (3)	C41—C42—H42	118.8
N4—C5—H5A	109.3	C44—C43—C42	120.3 (3)
C6—C5—H5A	109.3	C44—C43—H43	119.9
N4—C5—H5B	109.3	C42—C43—H43	119.9
C6—C5—H5B	109.3	O44—C44—C45	116.7 (3)
H5A—C5—H5B	108.0	O44—C44—C43	124.7 (3)
N1—C6—C5	110.9 (3)	C45—C44—C43	118.6 (3)
N1—C6—H6A	109.5	C46—C45—C44	121.1 (3)
C5—C6—H6A	109.5	C46—C45—H45	119.5
N1—C6—H6B	109.5	C44—C45—H45	119.5
C5—C6—H6B	109.5	C45—C46—C41	121.6 (3)
H6A—C6—H6B	108.0	C45—C46—H46	119.2
O17—C17—N1	121.9 (3)	C41—C46—H46	119.2
O17—C17—C11	119.3 (3)	C44—O44—C441	117.8 (3)
N1—C17—C11	118.7 (2)	O44—C441—H41A	109.5
C12—C11—C16	117.0 (3)	O44—C441—H41B	109.5
C12—C11—C17	121.1 (3)	H41A—C441—H41B	109.5
C16—C11—C17	121.4 (3)	O44—C441—H41C	109.5
F12—C12—C13	118.6 (3)	H41A—C441—H41C	109.5
F12—C12—C11	117.9 (3)	H41B—C441—H41C	109.5
			
C17—N1—C2—C3	116.2 (3)	C11—C12—C13—C14	1.0 (5)
C6—N1—C2—C3	−55.9 (4)	C12—C13—C14—C15	0.1 (5)
N1—C2—C3—N4	56.2 (4)	C13—C14—C15—C16	−1.0 (5)
C2—C3—N4—C41	169.4 (3)	C14—C15—C16—C11	0.9 (5)
C2—C3—N4—C5	−56.4 (4)	C12—C11—C16—C15	0.1 (4)
C41—N4—C5—C6	−170.6 (3)	C17—C11—C16—C15	−172.2 (3)
C3—N4—C5—C6	55.5 (4)	C5—N4—C41—C42	−176.2 (3)
C17—N1—C6—C5	−115.8 (4)	C3—N4—C41—C42	−44.8 (4)
C2—N1—C6—C5	55.9 (4)	C5—N4—C41—C46	5.7 (4)
N4—C5—C6—N1	−55.4 (4)	C3—N4—C41—C46	137.2 (3)
C6—N1—C17—O17	176.7 (3)	C46—C41—C42—C43	−0.3 (5)
C2—N1—C17—O17	5.7 (5)	N4—C41—C42—C43	−178.4 (3)
C6—N1—C17—C11	−5.5 (5)	C41—C42—C43—C44	0.5 (5)
C2—N1—C17—C11	−176.4 (3)	C42—C43—C44—O44	179.9 (3)
O17—C17—C11—C12	−81.2 (4)	C42—C43—C44—C45	0.6 (5)
N1—C17—C11—C12	100.9 (4)	O44—C44—C45—C46	178.8 (3)
O17—C17—C11—C16	90.8 (4)	C43—C44—C45—C46	−1.8 (5)
N1—C17—C11—C16	−87.2 (4)	C44—C45—C46—C41	2.1 (5)
C16—C11—C12—F12	179.6 (3)	C42—C41—C46—C45	−1.0 (5)
C17—C11—C12—F12	−8.1 (4)	N4—C41—C46—C45	177.1 (3)
C16—C11—C12—C13	−1.0 (5)	C45—C44—O44—C441	−175.1 (3)
C17—C11—C12—C13	171.2 (3)	C43—C44—O44—C441	5.5 (5)
F12—C12—C13—C14	−179.7 (3)		

### 1-(2-Fluorobenzoyl)-4-(4-methoxyphenyl)piperazine (II) Hydrogen-bond geometry (Å, º)

**Table:**

*D*—H···*A*	*D*—H	H···*A*	*D*···*A*	*D*—H···*A*
C2—H2*A*···O17^i^	0.97	2.50	3.387 (4)	152
C16—H16···O17^ii^	0.93	2.43	3.340 (5)	167

Symmetry codes: (i) −*x*+1, −*y*+1, −*z*+1; (ii) −*x*+2, −*y*+1, −*z*+1.

### 1-(2-Chlorobenzoyl)-4-(4-methoxyphenyl)piperazine (III) Crystal data

**Table:**

C_18_H_19_ClN_2_O_2_	*D*_x_ = 1.322 Mg m^−^^3^
*M**_r_* = 330.80	Mo *K*α radiation, λ = 0.71073 Å
Orthorhombic, *P**b**c**a*	Cell parameters from 3642 reflections
*a* = 13.0320 (11) Å	θ = 2.6–27.8°
*b* = 13.2470 (13) Å	µ = 0.24 mm^−^^1^
*c* = 19.258 (2) Å	*T* = 293 K
*V* = 3324.6 (6) Å^3^	Block, yellow
*Z* = 8	0.50 × 0.40 × 0.38 mm
*F*(000) = 1392	

### 1-(2-Chlorobenzoyl)-4-(4-methoxyphenyl)piperazine (III) Data collection

**Table:**

Oxford Diffraction Xcalibur diffractometer with Sapphire CCD	3642 independent reflections
Radiation source: Enhance (Mo) X-ray Source	2407 reflections with *I* > 2σ(*I*)
Graphite monochromator	*R*_int_ = 0.022
ω scans	θ_max_ = 27.8°, θ_min_ = 2.6°
Absorption correction: multi-scan (CrysAlis RED; Oxford Diffraction, 2009)	*h* = −16→17
*T*_min_ = 0.862, *T*_max_ = 0.912	*k* = −16→17
13862 measured reflections	*l* = −12→25

### 1-(2-Chlorobenzoyl)-4-(4-methoxyphenyl)piperazine (III) Refinement

**Table:**

Refinement on *F*^2^	26 restraints
Least-squares matrix: full	Hydrogen site location: inferred from neighbouring sites
*R*[*F*^2^ > 2σ(*F*^2^)] = 0.048	H-atom parameters constrained
*wR*(*F*^2^) = 0.127	*w* = 1/[σ^2^(*F*_o_^2^) + (0.0484*P*)^2^ + 1.4776*P*] where *P* = (*F*_o_^2^ + 2*F*_c_^2^)/3
*S* = 1.02	(Δ/σ)_max_ < 0.001
3642 reflections	Δρ_max_ = 0.23 e Å^−^^3^
243 parameters	Δρ_min_ = −0.45 e Å^−^^3^

### 1-(2-Chlorobenzoyl)-4-(4-methoxyphenyl)piperazine (III) Special details

**Table:**

Geometry. All esds (except the esd in the dihedral angle between two l.s. planes) are estimated using the full covariance matrix. The cell esds are taken into account individually in the estimation of esds in distances, angles and torsion angles; correlations between esds in cell parameters are only used when they are defined by crystal symmetry. An approximate (isotropic) treatment of cell esds is used for estimating esds involving l.s. planes.

### 1-(2-Chlorobenzoyl)-4-(4-methoxyphenyl)piperazine (III) Fractional atomic coordinates and isotropic or equivalent isotropic displacement parameters (Å^2^)

**Table:**

	*x*	*y*	*z*	*U*_iso_*/*U*_eq_	Occ. (<1)
N1	0.68382 (12)	0.28146 (13)	0.41176 (9)	0.0526 (4)	
C2	0.58246 (16)	0.25886 (18)	0.43972 (13)	0.0642 (6)	
H2A	0.5883	0.2424	0.4886	0.077*	
H2B	0.5542	0.2006	0.4160	0.077*	
C3	0.51117 (15)	0.34757 (17)	0.43087 (11)	0.0560 (6)	
H3A	0.4428	0.3292	0.4463	0.067*	
H3B	0.5349	0.4035	0.4592	0.067*	
N4	0.50753 (11)	0.37844 (12)	0.35839 (8)	0.0447 (4)	
C5	0.60999 (14)	0.40544 (16)	0.33358 (10)	0.0488 (5)	
H5A	0.6361	0.4619	0.3604	0.059*	
H5B	0.6061	0.4261	0.2853	0.059*	
C6	0.68172 (15)	0.31756 (17)	0.34028 (11)	0.0539 (5)	
H6A	0.6595	0.2635	0.3098	0.065*	
H6B	0.7502	0.3378	0.3263	0.065*	
C17	0.76756 (19)	0.26749 (18)	0.45027 (14)	0.0534 (6)	0.942 (2)
O17	0.76535 (19)	0.2354 (2)	0.50989 (12)	0.0936 (9)	0.942 (2)
C11	0.86962 (18)	0.29271 (17)	0.41824 (14)	0.0470 (5)	0.942 (2)
C12	0.9128 (2)	0.38720 (18)	0.42449 (16)	0.0535 (6)	0.942 (2)
Cl12	0.84046 (7)	0.48603 (5)	0.46012 (3)	0.0799 (3)	0.942 (2)
C13	1.0112 (3)	0.4066 (3)	0.4020 (2)	0.0726 (8)	0.942 (2)
H13	1.0393	0.4707	0.4073	0.087*	0.942 (2)
C14	1.0670 (3)	0.3318 (4)	0.3718 (3)	0.0882 (11)	0.942 (2)
H14	1.1339	0.3446	0.3573	0.106*	0.942 (2)
C15	1.0253 (3)	0.2376 (3)	0.36267 (19)	0.0843 (11)	0.942 (2)
H15	1.0628	0.1871	0.3407	0.101*	0.942 (2)
C16	0.9270 (2)	0.2185 (2)	0.38639 (15)	0.0670 (7)	0.942 (2)
H16	0.8991	0.1544	0.3808	0.080*	0.942 (2)
C37	0.773 (2)	0.2975 (19)	0.454 (2)	0.0534 (6)	0.058 (2)
O37	0.777 (3)	0.285 (4)	0.517 (2)	0.0936 (9)	0.058 (2)
C31	0.871 (3)	0.3261 (14)	0.418 (2)	0.0470 (5)	0.058 (2)
C32	0.935 (2)	0.2481 (15)	0.3977 (18)	0.0670 (7)	0.058 (2)
Cl32	0.8826 (19)	0.1267 (11)	0.4022 (13)	0.162 (10)	0.058 (2)
C33	1.035 (3)	0.264 (3)	0.379 (3)	0.0843 (11)	0.058 (2)
H33	1.0769	0.2102	0.3661	0.101*	0.058 (2)
C34	1.073 (3)	0.360 (3)	0.381 (5)	0.0882 (11)	0.058 (2)
H34	1.1407	0.3717	0.3675	0.106*	0.058 (2)
C35	1.013 (4)	0.439 (3)	0.403 (4)	0.0726 (8)	0.058 (2)
H35	1.0396	0.5039	0.4036	0.087*	0.058 (2)
C36	0.913 (3)	0.4206 (16)	0.424 (3)	0.0535 (6)	0.058 (2)
H36	0.8746	0.4729	0.4429	0.064*	0.058 (2)
C41	0.42740 (14)	0.44424 (14)	0.33779 (10)	0.0415 (4)	
C42	0.35531 (15)	0.48382 (16)	0.38335 (11)	0.0523 (5)	
H42	0.3599	0.4687	0.4304	0.063*	
C43	0.27626 (15)	0.54574 (17)	0.35992 (11)	0.0547 (5)	
H43	0.2290	0.5716	0.3914	0.066*	
C44	0.26749 (14)	0.56901 (15)	0.29086 (10)	0.0469 (5)	
C45	0.33768 (16)	0.52910 (16)	0.24458 (11)	0.0527 (5)	
H45	0.3323	0.5440	0.1975	0.063*	
C46	0.41523 (16)	0.46762 (16)	0.26773 (10)	0.0519 (5)	
H46	0.4611	0.4407	0.2357	0.062*	
O44	0.19396 (11)	0.62997 (12)	0.26191 (8)	0.0676 (5)	
C441	0.1267 (2)	0.6808 (2)	0.30737 (15)	0.0838 (8)	
H41A	0.0850	0.7271	0.2814	0.126*	
H41B	0.1658	0.7173	0.3413	0.126*	
H41C	0.0834	0.6325	0.3303	0.126*	

### 1-(2-Chlorobenzoyl)-4-(4-methoxyphenyl)piperazine (III) Atomic displacement parameters (Å^2^)

**Table:**

	*U*^11^	*U*^22^	*U*^33^	*U*^12^	*U*^13^	*U*^23^
N1	0.0402 (9)	0.0602 (11)	0.0574 (10)	0.0018 (8)	−0.0043 (8)	0.0178 (9)
C2	0.0463 (12)	0.0702 (15)	0.0760 (15)	−0.0061 (11)	−0.0039 (11)	0.0334 (12)
C3	0.0438 (11)	0.0697 (14)	0.0546 (12)	−0.0020 (10)	0.0042 (9)	0.0198 (11)
N4	0.0396 (8)	0.0489 (9)	0.0455 (9)	0.0011 (7)	0.0009 (7)	0.0096 (7)
C5	0.0410 (10)	0.0582 (12)	0.0472 (11)	0.0023 (10)	0.0022 (8)	0.0129 (9)
C6	0.0442 (11)	0.0664 (14)	0.0510 (12)	0.0062 (10)	−0.0026 (9)	0.0051 (10)
C17	0.0481 (12)	0.0511 (14)	0.0610 (14)	0.0011 (12)	−0.0065 (10)	0.0135 (12)
O17	0.0598 (12)	0.147 (2)	0.0735 (12)	−0.0024 (16)	−0.0080 (9)	0.0564 (16)
C11	0.0435 (11)	0.0495 (13)	0.0482 (11)	0.0035 (12)	−0.0107 (9)	0.0073 (12)
C12	0.0615 (13)	0.0566 (15)	0.0424 (11)	−0.0085 (15)	−0.0129 (10)	0.0079 (13)
Cl12	0.1176 (7)	0.0594 (4)	0.0626 (4)	−0.0059 (4)	−0.0025 (4)	−0.0119 (3)
C13	0.0680 (16)	0.092 (2)	0.0575 (14)	−0.0303 (18)	−0.0207 (12)	0.024 (2)
C14	0.0466 (14)	0.143 (3)	0.075 (3)	0.000 (2)	−0.0060 (14)	0.041 (2)
C15	0.0634 (17)	0.113 (3)	0.076 (3)	0.0371 (19)	0.0043 (16)	0.0098 (19)
C16	0.0605 (15)	0.0611 (17)	0.0794 (18)	0.0121 (14)	−0.0092 (13)	−0.0010 (14)
C37	0.0481 (12)	0.0511 (14)	0.0610 (14)	0.0011 (12)	−0.0065 (10)	0.0135 (12)
O37	0.0598 (12)	0.147 (2)	0.0735 (12)	−0.0024 (16)	−0.0080 (9)	0.0564 (16)
C31	0.0435 (11)	0.0495 (13)	0.0482 (11)	0.0035 (12)	−0.0107 (9)	0.0073 (12)
C32	0.0605 (15)	0.0611 (17)	0.0794 (18)	0.0121 (14)	−0.0092 (13)	−0.0010 (14)
Cl32	0.22 (2)	0.072 (9)	0.19 (2)	0.000 (11)	−0.011 (17)	−0.030 (11)
C33	0.0634 (17)	0.113 (3)	0.076 (3)	0.0371 (19)	0.0043 (16)	0.0098 (19)
C34	0.0466 (14)	0.143 (3)	0.075 (3)	0.000 (2)	−0.0060 (14)	0.041 (2)
C35	0.0680 (16)	0.092 (2)	0.0575 (14)	−0.0303 (18)	−0.0207 (12)	0.024 (2)
C36	0.0615 (13)	0.0566 (15)	0.0424 (11)	−0.0085 (15)	−0.0129 (10)	0.0079 (13)
C41	0.0383 (9)	0.0408 (10)	0.0454 (10)	−0.0011 (8)	0.0000 (8)	0.0008 (8)
C42	0.0483 (11)	0.0649 (13)	0.0436 (11)	0.0045 (10)	0.0057 (9)	0.0076 (10)
C43	0.0422 (11)	0.0633 (14)	0.0586 (13)	0.0070 (10)	0.0122 (9)	0.0011 (10)
C44	0.0411 (10)	0.0433 (11)	0.0563 (12)	0.0035 (9)	−0.0017 (9)	−0.0012 (9)
C45	0.0588 (12)	0.0577 (12)	0.0417 (10)	0.0126 (11)	−0.0039 (9)	−0.0020 (9)
C46	0.0523 (12)	0.0603 (13)	0.0431 (11)	0.0163 (10)	0.0025 (9)	−0.0064 (9)
O44	0.0592 (9)	0.0722 (10)	0.0715 (10)	0.0277 (8)	−0.0029 (8)	0.0013 (8)
C441	0.0720 (16)	0.0823 (18)	0.097 (2)	0.0365 (15)	0.0069 (15)	0.0016 (16)

### 1-(2-Chlorobenzoyl)-4-(4-methoxyphenyl)piperazine (III) Geometric parameters (Å, º)

**Table:**

N1—C17	1.332 (3)	C16—H16	0.9300
N1—C37	1.43 (4)	C37—O37	1.222 (10)
N1—C2	1.458 (3)	C37—C31	1.508 (9)
N1—C6	1.458 (3)	C31—C36	1.372 (10)
C2—C3	1.508 (3)	C31—C32	1.381 (9)
C2—H2A	0.9700	C32—C33	1.371 (10)
C2—H2B	0.9700	C32—Cl32	1.749 (10)
C3—N4	1.455 (2)	C33—C34	1.362 (10)
C3—H3A	0.9700	C33—H33	0.9300
C3—H3B	0.9700	C34—C35	1.372 (11)
N4—C41	1.417 (2)	C34—H34	0.9300
N4—C5	1.463 (2)	C35—C36	1.385 (10)
C5—C6	1.499 (3)	C35—H35	0.9300
C5—H5A	0.9700	C36—H36	0.9300
C5—H5B	0.9700	C41—C42	1.388 (3)
C6—H6A	0.9700	C41—C46	1.393 (3)
C6—H6B	0.9700	C42—C43	1.392 (3)
C17—O17	1.225 (3)	C42—H42	0.9300
C17—C11	1.504 (3)	C43—C44	1.370 (3)
C11—C12	1.378 (3)	C43—H43	0.9300
C11—C16	1.379 (3)	C44—O44	1.372 (2)
C12—C13	1.378 (4)	C44—C45	1.382 (3)
C12—Cl12	1.753 (3)	C45—C46	1.372 (3)
C13—C14	1.359 (4)	C45—H45	0.9300
C13—H13	0.9300	C46—H46	0.9300
C14—C15	1.373 (5)	O44—C441	1.410 (3)
C14—H14	0.9300	C441—H41A	0.9600
C15—C16	1.384 (4)	C441—H41B	0.9600
C15—H15	0.9300	C441—H41C	0.9600
			
C17—N1—C2	120.55 (18)	C11—C16—C15	121.2 (3)
C37—N1—C2	123.8 (13)	C11—C16—H16	119.4
C17—N1—C6	125.92 (18)	C15—C16—H16	119.4
C37—N1—C6	120.1 (13)	O37—C37—N1	125 (3)
C2—N1—C6	113.53 (16)	O37—C37—C31	116.5 (17)
N1—C2—C3	110.89 (17)	N1—C37—C31	118 (3)
N1—C2—H2A	109.5	C36—C31—C32	117.9 (11)
C3—C2—H2A	109.5	C36—C31—C37	122.0 (14)
N1—C2—H2B	109.5	C32—C31—C37	116.9 (12)
C3—C2—H2B	109.5	C33—C32—C31	122.1 (11)
H2A—C2—H2B	108.0	C33—C32—Cl32	121.7 (12)
N4—C3—C2	110.33 (18)	C31—C32—Cl32	116.1 (11)
N4—C3—H3A	109.6	C34—C33—C32	118.9 (13)
C2—C3—H3A	109.6	C34—C33—H33	120.6
N4—C3—H3B	109.6	C32—C33—H33	120.6
C2—C3—H3B	109.6	C33—C34—C35	120.7 (13)
H3A—C3—H3B	108.1	C33—C34—H34	119.7
C41—N4—C3	117.74 (15)	C35—C34—H34	119.7
C41—N4—C5	115.52 (14)	C34—C35—C36	119.6 (13)
C3—N4—C5	110.63 (15)	C34—C35—H35	120.2
N4—C5—C6	110.57 (17)	C36—C35—H35	120.2
N4—C5—H5A	109.5	C31—C36—C35	120.5 (13)
C6—C5—H5A	109.5	C31—C36—H36	119.8
N4—C5—H5B	109.5	C35—C36—H36	119.8
C6—C5—H5B	109.5	C42—C41—C46	116.81 (17)
H5A—C5—H5B	108.1	C42—C41—N4	123.64 (17)
N1—C6—C5	110.36 (17)	C46—C41—N4	119.46 (16)
N1—C6—H6A	109.6	C41—C42—C43	121.23 (19)
C5—C6—H6A	109.6	C41—C42—H42	119.4
N1—C6—H6B	109.6	C43—C42—H42	119.4
C5—C6—H6B	109.6	C44—C43—C42	120.60 (18)
H6A—C6—H6B	108.1	C44—C43—H43	119.7
O17—C17—N1	123.4 (2)	C42—C43—H43	119.7
O17—C17—C11	118.9 (2)	C43—C44—O44	125.84 (18)
N1—C17—C11	117.7 (2)	C43—C44—C45	118.99 (18)
C12—C11—C16	117.7 (2)	O44—C44—C45	115.18 (18)
C12—C11—C17	121.8 (2)	C46—C45—C44	120.32 (19)
C16—C11—C17	120.2 (2)	C46—C45—H45	119.8
C13—C12—C11	121.5 (3)	C44—C45—H45	119.8
C13—C12—Cl12	119.0 (2)	C45—C46—C41	122.02 (18)
C11—C12—Cl12	119.53 (19)	C45—C46—H46	119.0
C14—C13—C12	119.8 (3)	C41—C46—H46	119.0
C14—C13—H13	120.1	C44—O44—C441	117.58 (18)
C12—C13—H13	120.1	O44—C441—H41A	109.5
C13—C14—C15	120.4 (3)	O44—C441—H41B	109.5
C13—C14—H14	119.8	H41A—C441—H41B	109.5
C15—C14—H14	119.8	O44—C441—H41C	109.5
C14—C15—C16	119.4 (3)	H41A—C441—H41C	109.5
C14—C15—H15	120.3	H41B—C441—H41C	109.5
C16—C15—H15	120.3		
			
C17—N1—C2—C3	127.9 (2)	C6—N1—C37—O37	168 (2)
C37—N1—C2—C3	108.7 (12)	C17—N1—C37—C31	99 (5)
C6—N1—C2—C3	−52.9 (3)	C2—N1—C37—C31	−176.6 (10)
N1—C2—C3—N4	54.7 (3)	C6—N1—C37—C31	−16 (2)
C2—C3—N4—C41	165.51 (16)	O37—C37—C31—C36	−73 (4)
C2—C3—N4—C5	−58.6 (2)	N1—C37—C31—C36	110 (3)
C41—N4—C5—C6	−163.56 (16)	O37—C37—C31—C32	86 (3)
C3—N4—C5—C6	59.5 (2)	N1—C37—C31—C32	−90 (3)
C17—N1—C6—C5	−127.5 (2)	C36—C31—C32—C33	−5 (2)
C37—N1—C6—C5	−109.0 (12)	C37—C31—C32—C33	−165 (3)
C2—N1—C6—C5	53.4 (2)	C36—C31—C32—Cl32	171 (3)
N4—C5—C6—N1	−55.8 (2)	C37—C31—C32—Cl32	11 (4)
C37—N1—C17—O17	107 (5)	C31—C32—C33—C34	0 (3)
C2—N1—C17—O17	0.2 (4)	Cl32—C32—C33—C34	−176 (4)
C6—N1—C17—O17	−178.8 (2)	C32—C33—C34—C35	2 (6)
C37—N1—C17—C11	−73 (5)	C33—C34—C35—C36	1 (8)
C2—N1—C17—C11	−179.8 (2)	C32—C31—C36—C35	8 (5)
C6—N1—C17—C11	1.2 (3)	C37—C31—C36—C35	167 (5)
O17—C17—C11—C12	−89.1 (3)	C34—C35—C36—C31	−6 (8)
N1—C17—C11—C12	90.9 (3)	C3—N4—C41—C42	2.8 (3)
O17—C17—C11—C16	85.4 (3)	C5—N4—C41—C42	−131.0 (2)
N1—C17—C11—C16	−94.6 (3)	C3—N4—C41—C46	−173.72 (19)
C16—C11—C12—C13	−2.1 (4)	C5—N4—C41—C46	52.5 (2)
C17—C11—C12—C13	172.5 (3)	C46—C41—C42—C43	−1.4 (3)
C16—C11—C12—Cl12	176.4 (2)	N4—C41—C42—C43	−178.03 (18)
C17—C11—C12—Cl12	−8.9 (3)	C41—C42—C43—C44	0.2 (3)
C11—C12—C13—C14	1.0 (5)	C42—C43—C44—O44	−179.13 (19)
Cl12—C12—C13—C14	−177.5 (3)	C42—C43—C44—C45	0.7 (3)
C12—C13—C14—C15	1.1 (5)	C43—C44—C45—C46	−0.4 (3)
C13—C14—C15—C16	−2.1 (5)	O44—C44—C45—C46	179.50 (19)
C12—C11—C16—C15	1.2 (4)	C44—C45—C46—C41	−0.9 (3)
C17—C11—C16—C15	−173.6 (3)	C42—C41—C46—C45	1.8 (3)
C14—C15—C16—C11	0.9 (4)	N4—C41—C46—C45	178.55 (19)
C17—N1—C37—O37	−76 (5)	C43—C44—O44—C441	6.1 (3)
C2—N1—C37—O37	7 (3)	C45—C44—O44—C441	−173.8 (2)

### 1-(2-Chlorobenzoyl)-4-(4-methoxyphenyl)piperazine (III) Hydrogen-bond geometry (Å, º)

**Table:**

*D*—H···*A*	*D*—H	H···*A*	*D*···*A*	*D*—H···*A*
C3—H3*A*···O17^i^	0.97	2.61	3.574 (3)	175
C2—H2*A*···*Cg*1^i^	0.97	2.84	3.648 (3)	142
C15—H15···*Cg*2^ii^	0.93	2.72	3.610 (4)	161

Symmetry codes: (i) *x*−1/2, −*y*+1/2, −*z*+1; (ii) −*x*+3/2, *y*−1/2, *z*.

### 1-(2-Bromoobenzoyl)-4-(4-methoxyphenyl)piperazine (IV) Crystal data

**Table:**

C_18_H_19_BrN_2_O_2_	*D*_x_ = 1.481 Mg m^−^^3^
*M**_r_* = 375.26	Mo *K*α radiation, λ = 0.71073 Å
Orthorhombic, *P**b**c**a*	Cell parameters from 10112 reflections
*a* = 12.9119 (14) Å	θ = 2.2–30.6°
*b* = 13.3664 (16) Å	µ = 2.45 mm^−^^1^
*c* = 19.5019 (19) Å	*T* = 293 K
*V* = 3365.7 (6) Å^3^	Block, colourless
*Z* = 8	0.22 × 0.21 × 0.18 mm
*F*(000) = 1536	

### 1-(2-Bromoobenzoyl)-4-(4-methoxyphenyl)piperazine (IV) Data collection

**Table:**

Bruker D8 Quest diffractometer	4262 independent reflections
Radiation source: Enhance (Mo) X-ray Source	3135 reflections with *I* > 2σ(*I*)
Graphite monochromator	*R*_int_ = 0.039
φ and ω scans	θ_max_ = 28.6°, θ_min_ = 2.4°
Absorption correction: multi-scan (SADABS; Bruker, 2015	*h* = −17→17
*T*_min_ = 0.538, *T*_max_ = 0.643	*k* = −17→17
47663 measured reflections	*l* = −25→26

### 1-(2-Bromoobenzoyl)-4-(4-methoxyphenyl)piperazine (IV) Refinement

**Table:**

Refinement on *F*^2^	Primary atom site location: difference Fourier map
Least-squares matrix: full	Hydrogen site location: inferred from neighbouring sites
*R*[*F*^2^ > 2σ(*F*^2^)] = 0.045	H-atom parameters constrained
*wR*(*F*^2^) = 0.131	*w* = 1/[σ^2^(*F*_o_^2^) + (0.0626*P*)^2^ + 2.0275*P*] where *P* = (*F*_o_^2^ + 2*F*_c_^2^)/3
*S* = 1.02	(Δ/σ)_max_ = 0.001
4262 reflections	Δρ_max_ = 0.54 e Å^−^^3^
209 parameters	Δρ_min_ = −0.64 e Å^−^^3^
0 restraints	

### 1-(2-Bromoobenzoyl)-4-(4-methoxyphenyl)piperazine (IV) Special details

**Table:**

Geometry. All esds (except the esd in the dihedral angle between two l.s. planes) are estimated using the full covariance matrix. The cell esds are taken into account individually in the estimation of esds in distances, angles and torsion angles; correlations between esds in cell parameters are only used when they are defined by crystal symmetry. An approximate (isotropic) treatment of cell esds is used for estimating esds involving l.s. planes.

### 1-(2-Bromoobenzoyl)-4-(4-methoxyphenyl)piperazine (IV) Fractional atomic coordinates and isotropic or equivalent isotropic displacement parameters (Å^2^)

**Table:**

	*x*	*y*	*z*	*U*_iso_*/*U*_eq_	
N1	0.68078 (15)	0.28092 (16)	0.41388 (11)	0.0487 (5)	
C2	0.57868 (19)	0.2622 (2)	0.44328 (15)	0.0590 (7)	
H2A	0.5856	0.2493	0.4920	0.071*	
H2B	0.5487	0.2032	0.4221	0.071*	
C3	0.50766 (18)	0.3504 (2)	0.43240 (12)	0.0519 (6)	
H3A	0.4388	0.3340	0.4487	0.062*	
H3B	0.5328	0.4072	0.4584	0.062*	
N4	0.50316 (13)	0.37615 (15)	0.35983 (9)	0.0419 (4)	
C5	0.60654 (17)	0.4002 (2)	0.33390 (12)	0.0473 (5)	
H5A	0.6341	0.4572	0.3587	0.057*	
H5B	0.6021	0.4180	0.2858	0.057*	
C6	0.67798 (19)	0.3122 (2)	0.34234 (12)	0.0521 (6)	
H6A	0.6541	0.2572	0.3140	0.062*	
H6B	0.7471	0.3304	0.3274	0.062*	
C17	0.76628 (19)	0.2666 (2)	0.45102 (13)	0.0512 (6)	
O17	0.76512 (15)	0.2365 (2)	0.50994 (12)	0.0946 (9)	
C11	0.86917 (17)	0.28780 (19)	0.41712 (12)	0.0454 (5)	
C12	0.91561 (19)	0.3799 (2)	0.42084 (11)	0.0497 (6)	
Br12	0.84357 (3)	0.49042 (2)	0.45966 (2)	0.07101 (15)	
C13	1.0151 (2)	0.3952 (3)	0.39598 (13)	0.0650 (8)	
H13	1.0461	0.4579	0.3993	0.078*	
C14	1.0668 (2)	0.3174 (4)	0.36663 (16)	0.0789 (11)	
H14	1.1339	0.3267	0.3504	0.095*	
C15	1.0212 (3)	0.2262 (3)	0.36096 (17)	0.0798 (10)	
H15	1.0563	0.1741	0.3396	0.096*	
C16	0.9229 (2)	0.2107 (2)	0.38671 (15)	0.0650 (7)	
H16	0.8928	0.1477	0.3835	0.078*	
C41	0.42268 (16)	0.44128 (16)	0.33883 (11)	0.0390 (5)	
C42	0.35098 (18)	0.4829 (2)	0.38304 (13)	0.0491 (6)	
H42	0.3559	0.4695	0.4297	0.059*	
C43	0.27162 (18)	0.5446 (2)	0.35948 (13)	0.0527 (6)	
H43	0.2243	0.5715	0.3903	0.063*	
C44	0.26318 (17)	0.56563 (19)	0.29071 (11)	0.0450 (5)	
C45	0.3328 (2)	0.5236 (2)	0.24572 (13)	0.0527 (6)	
H45	0.3271	0.5368	0.1990	0.063*	
C46	0.41060 (19)	0.4627 (2)	0.26907 (12)	0.0498 (6)	
H46	0.4565	0.4348	0.2377	0.060*	
O44	0.18982 (15)	0.62631 (15)	0.26166 (10)	0.0658 (5)	
C441	0.1221 (3)	0.6779 (3)	0.30621 (19)	0.0798 (10)	
H41A	0.0799	0.7232	0.2801	0.120*	
H41B	0.1618	0.7148	0.3392	0.120*	
H41C	0.0785	0.6307	0.3295	0.120*	

### 1-(2-Bromoobenzoyl)-4-(4-methoxyphenyl)piperazine (IV) Atomic displacement parameters (Å^2^)

**Table:**

	*U*^11^	*U*^22^	*U*^33^	*U*^12^	*U*^13^	*U*^23^
N1	0.0384 (10)	0.0551 (12)	0.0527 (11)	0.0020 (9)	−0.0042 (8)	0.0126 (9)
C2	0.0415 (13)	0.0652 (17)	0.0704 (16)	−0.0037 (12)	−0.0026 (12)	0.0291 (14)
C3	0.0411 (12)	0.0667 (16)	0.0478 (12)	0.0014 (12)	0.0032 (10)	0.0187 (12)
N4	0.0346 (9)	0.0492 (11)	0.0420 (9)	0.0031 (8)	−0.0005 (7)	0.0078 (8)
C5	0.0384 (11)	0.0606 (15)	0.0429 (11)	0.0028 (11)	0.0015 (9)	0.0121 (11)
C6	0.0410 (12)	0.0690 (17)	0.0462 (12)	0.0091 (12)	−0.0034 (10)	0.0019 (12)
C17	0.0444 (13)	0.0526 (14)	0.0566 (14)	0.0029 (11)	−0.0065 (10)	0.0121 (11)
O17	0.0535 (11)	0.158 (3)	0.0728 (14)	0.0043 (14)	−0.0081 (10)	0.0598 (15)
C11	0.0395 (11)	0.0524 (14)	0.0442 (12)	0.0042 (10)	−0.0099 (9)	0.0036 (10)
C12	0.0519 (13)	0.0615 (15)	0.0357 (11)	−0.0014 (12)	−0.0091 (9)	0.0028 (10)
Br12	0.1004 (3)	0.0570 (2)	0.0557 (2)	−0.00293 (15)	−0.00205 (15)	−0.01036 (12)
C13	0.0542 (15)	0.091 (2)	0.0494 (14)	−0.0214 (16)	−0.0125 (12)	0.0166 (14)
C14	0.0434 (15)	0.134 (3)	0.0594 (17)	0.0089 (19)	−0.0024 (13)	0.025 (2)
C15	0.0631 (19)	0.107 (3)	0.070 (2)	0.034 (2)	−0.0001 (15)	0.0001 (19)
C16	0.0592 (16)	0.0640 (17)	0.0720 (18)	0.0157 (14)	−0.0086 (13)	−0.0041 (14)
C41	0.0361 (10)	0.0400 (11)	0.0409 (11)	−0.0010 (9)	−0.0020 (8)	0.0017 (9)
C42	0.0418 (12)	0.0658 (16)	0.0398 (12)	0.0062 (11)	0.0061 (9)	0.0092 (11)
C43	0.0413 (13)	0.0646 (16)	0.0520 (13)	0.0094 (12)	0.0085 (10)	0.0035 (12)
C44	0.0380 (11)	0.0454 (13)	0.0514 (12)	0.0051 (10)	−0.0051 (9)	−0.0012 (10)
C45	0.0575 (15)	0.0621 (15)	0.0386 (11)	0.0137 (12)	−0.0058 (10)	−0.0035 (11)
C46	0.0512 (13)	0.0608 (15)	0.0373 (11)	0.0171 (12)	−0.0018 (10)	−0.0071 (10)
O44	0.0611 (11)	0.0735 (13)	0.0629 (11)	0.0303 (10)	−0.0045 (9)	0.0034 (10)
C441	0.0693 (19)	0.081 (2)	0.089 (2)	0.0359 (18)	0.0017 (17)	−0.0007 (18)

### 1-(2-Bromoobenzoyl)-4-(4-methoxyphenyl)piperazine (IV) Geometric parameters (Å, º)

**Table:**

N1—C17	1.334 (3)	C13—C14	1.363 (5)
N1—C6	1.457 (3)	C13—H13	0.9300
N1—C2	1.459 (3)	C14—C15	1.358 (5)
C2—C3	1.508 (4)	C14—H14	0.9300
C2—H2A	0.9700	C15—C16	1.381 (5)
C2—H2B	0.9700	C15—H15	0.9300
C3—N4	1.458 (3)	C16—H16	0.9300
C3—H3A	0.9700	C41—C42	1.382 (3)
C3—H3B	0.9700	C41—C46	1.399 (3)
N4—C41	1.416 (3)	C42—C43	1.393 (3)
N4—C5	1.463 (3)	C42—H42	0.9300
C5—C6	1.504 (3)	C43—C44	1.375 (3)
C5—H5A	0.9700	C43—H43	0.9300
C5—H5B	0.9700	C44—O44	1.370 (3)
C6—H6A	0.9700	C44—C45	1.376 (3)
C6—H6B	0.9700	C45—C46	1.371 (4)
C17—O17	1.218 (3)	C45—H45	0.9300
C17—C11	1.511 (3)	C46—H46	0.9300
C11—C12	1.371 (4)	O44—C441	1.413 (4)
C11—C16	1.377 (4)	C441—H41A	0.9600
C12—C13	1.389 (4)	C441—H41B	0.9600
C12—Br12	1.903 (3)	C441—H41C	0.9600
			
C17—N1—C6	125.6 (2)	C13—C12—Br12	118.4 (2)
C17—N1—C2	120.6 (2)	C14—C13—C12	119.2 (3)
C6—N1—C2	113.77 (19)	C14—C13—H13	120.4
N1—C2—C3	111.1 (2)	C12—C13—H13	120.4
N1—C2—H2A	109.4	C15—C14—C13	120.4 (3)
C3—C2—H2A	109.4	C15—C14—H14	119.8
N1—C2—H2B	109.4	C13—C14—H14	119.8
C3—C2—H2B	109.4	C14—C15—C16	120.3 (3)
H2A—C2—H2B	108.0	C14—C15—H15	119.9
N4—C3—C2	110.2 (2)	C16—C15—H15	119.9
N4—C3—H3A	109.6	C11—C16—C15	120.5 (3)
C2—C3—H3A	109.6	C11—C16—H16	119.8
N4—C3—H3B	109.6	C15—C16—H16	119.8
C2—C3—H3B	109.6	C42—C41—C46	116.7 (2)
H3A—C3—H3B	108.1	C42—C41—N4	124.0 (2)
C41—N4—C3	117.09 (18)	C46—C41—N4	119.25 (19)
C41—N4—C5	115.76 (18)	C41—C42—C43	121.7 (2)
C3—N4—C5	110.56 (17)	C41—C42—H42	119.2
N4—C5—C6	110.5 (2)	C43—C42—H42	119.2
N4—C5—H5A	109.6	C44—C43—C42	120.1 (2)
C6—C5—H5A	109.6	C44—C43—H43	120.0
N4—C5—H5B	109.6	C42—C43—H43	120.0
C6—C5—H5B	109.6	O44—C44—C43	125.4 (2)
H5A—C5—H5B	108.1	O44—C44—C45	115.4 (2)
N1—C6—C5	110.1 (2)	C43—C44—C45	119.1 (2)
N1—C6—H6A	109.6	C46—C45—C44	120.6 (2)
C5—C6—H6A	109.6	C46—C45—H45	119.7
N1—C6—H6B	109.6	C44—C45—H45	119.7
C5—C6—H6B	109.6	C45—C46—C41	121.7 (2)
H6A—C6—H6B	108.1	C45—C46—H46	119.1
O17—C17—N1	123.3 (2)	C41—C46—H46	119.1
O17—C17—C11	119.1 (2)	C44—O44—C441	117.6 (2)
N1—C17—C11	117.6 (2)	O44—C441—H41A	109.5
C12—C11—C16	118.3 (2)	O44—C441—H41B	109.5
C12—C11—C17	122.0 (2)	H41A—C441—H41B	109.5
C16—C11—C17	119.4 (2)	O44—C441—H41C	109.5
C11—C12—C13	121.3 (3)	H41A—C441—H41C	109.5
C11—C12—Br12	120.29 (19)	H41B—C441—H41C	109.5
			
C17—N1—C2—C3	129.3 (3)	Br12—C12—C13—C14	−178.5 (2)
C6—N1—C2—C3	−52.6 (3)	C12—C13—C14—C15	0.8 (4)
N1—C2—C3—N4	54.3 (3)	C13—C14—C15—C16	−1.9 (5)
C2—C3—N4—C41	165.8 (2)	C12—C11—C16—C15	0.2 (4)
C2—C3—N4—C5	−58.7 (3)	C17—C11—C16—C15	−174.1 (3)
C41—N4—C5—C6	−163.87 (19)	C14—C15—C16—C11	1.4 (5)
C3—N4—C5—C6	60.0 (3)	C3—N4—C41—C42	1.7 (3)
C17—N1—C6—C5	−128.8 (3)	C5—N4—C41—C42	−131.6 (2)
C2—N1—C6—C5	53.1 (3)	C3—N4—C41—C46	−175.5 (2)
N4—C5—C6—N1	−56.0 (3)	C5—N4—C41—C46	51.3 (3)
C6—N1—C17—O17	−176.9 (3)	C46—C41—C42—C43	−1.1 (4)
C2—N1—C17—O17	1.1 (4)	N4—C41—C42—C43	−178.3 (2)
C6—N1—C17—C11	2.2 (4)	C41—C42—C43—C44	−0.2 (4)
C2—N1—C17—C11	−179.9 (2)	C42—C43—C44—O44	−178.8 (2)
O17—C17—C11—C12	−89.6 (3)	C42—C43—C44—C45	1.2 (4)
N1—C17—C11—C12	91.3 (3)	O44—C44—C45—C46	179.1 (3)
O17—C17—C11—C16	84.4 (4)	C43—C44—C45—C46	−0.9 (4)
N1—C17—C11—C16	−94.7 (3)	C44—C45—C46—C41	−0.4 (4)
C16—C11—C12—C13	−1.3 (4)	C42—C41—C46—C45	1.4 (4)
C17—C11—C12—C13	172.8 (2)	N4—C41—C46—C45	178.7 (2)
C16—C11—C12—Br12	178.01 (19)	C43—C44—O44—C441	5.3 (4)
C17—C11—C12—Br12	−7.9 (3)	C45—C44—O44—C441	−174.7 (3)
C11—C12—C13—C14	0.8 (4)		

### 1-(2-Bromoobenzoyl)-4-(4-methoxyphenyl)piperazine (IV) Hydrogen-bond geometry (Å, º)

**Table:**

*D*—H···*A*	*D*—H	H···*A*	*D*···*A*	*D*—H···*A*
C3—H3*A*···O17^i^	0.97	2.56	3.524 (3)	171
C2—H2*A*···*Cg*1^i^	0.97	2.82	3.630 (3)	142
C15—H15···*Cg*2^ii^	0.93	2.68	3.579 (4)	164

Symmetry codes: (i) *x*−1/2, −*y*+1/2, −*z*+1; (ii) −*x*+3/2, *y*−1/2, *z*.

### 1-(2-Iodobenzoyl)-4-(4-methoxyphenyl)piperazine (V) Crystal data

**Table:**

C_18_H_19_IN_2_O_2_	*D*_x_ = 1.602 Mg m^−^^3^
*M**_r_* = 422.25	Mo *K*α radiation, λ = 0.71073 Å
Orthorhombic, *P**b**c**a*	Cell parameters from 3838 reflections
*a* = 12.7671 (13) Å	θ = 2.6–27.7°
*b* = 13.5429 (12) Å	µ = 1.84 mm^−^^1^
*c* = 20.2542 (16) Å	*T* = 293 K
*V* = 3502.0 (5) Å^3^	Block, orange
*Z* = 8	0.48 × 0.42 × 0.38 mm
*F*(000) = 1680	

### 1-(2-Iodobenzoyl)-4-(4-methoxyphenyl)piperazine (V) Data collection

**Table:**

Oxford Diffraction Xcalibur diffractometer with Sapphire CCD	3838 independent reflections
Radiation source: Enhance (Mo) X-ray Source	3062 reflections with *I* > 2σ(*I*)
Graphite monochromator	*R*_int_ = 0.029
ω scans	θ_max_ = 27.7°, θ_min_ = 2.6°
Absorption correction: multi-scan (CrysAlis RED; Oxford Diffraction, 2009)	*h* = −16→9
*T*_min_ = 0.408, *T*_max_ = 0.497	*k* = −17→12
14215 measured reflections	*l* = −25→26

### 1-(2-Iodobenzoyl)-4-(4-methoxyphenyl)piperazine (V) Refinement

**Table:**

Refinement on *F*^2^	Primary atom site location: difference Fourier map
Least-squares matrix: full	Hydrogen site location: inferred from neighbouring sites
*R*[*F*^2^ > 2σ(*F*^2^)] = 0.068	H-atom parameters constrained
*wR*(*F*^2^) = 0.146	*w* = 1/[σ^2^(*F*_o_^2^) + 37.1584*P*] where *P* = (*F*_o_^2^ + 2*F*_c_^2^)/3
*S* = 1.18	(Δ/σ)_max_ < 0.001
3838 reflections	Δρ_max_ = 1.27 e Å^−^^3^
209 parameters	Δρ_min_ = −2.19 e Å^−^^3^
0 restraints	

### 1-(2-Iodobenzoyl)-4-(4-methoxyphenyl)piperazine (V) Special details

**Table:**

Geometry. All esds (except the esd in the dihedral angle between two l.s. planes) are estimated using the full covariance matrix. The cell esds are taken into account individually in the estimation of esds in distances, angles and torsion angles; correlations between esds in cell parameters are only used when they are defined by crystal symmetry. An approximate (isotropic) treatment of cell esds is used for estimating esds involving l.s. planes.

### 1-(2-Iodobenzoyl)-4-(4-methoxyphenyl)piperazine (V) Fractional atomic coordinates and isotropic or equivalent isotropic displacement parameters (Å^2^)

**Table:**

	*x*	*y*	*z*	*U*_iso_*/*U*_eq_	
N1	0.6767 (5)	0.2532 (5)	0.4083 (3)	0.0444 (15)	
C2	0.5718 (6)	0.2371 (7)	0.4337 (5)	0.061 (3)	
H2A	0.5755	0.2250	0.4808	0.073*	
H2B	0.5421	0.1789	0.4130	0.073*	
C3	0.5017 (6)	0.3231 (7)	0.4213 (4)	0.052 (2)	
H3A	0.4312	0.3075	0.4358	0.062*	
H3B	0.5261	0.3796	0.4465	0.062*	
N4	0.5006 (4)	0.3478 (4)	0.3506 (3)	0.0390 (14)	
C5	0.6074 (5)	0.3718 (6)	0.3295 (4)	0.0445 (18)	
H5A	0.6329	0.4278	0.3545	0.053*	
H5B	0.6070	0.3899	0.2831	0.053*	
C6	0.6796 (6)	0.2846 (6)	0.3397 (4)	0.0453 (18)	
H6A	0.6582	0.2305	0.3114	0.054*	
H6B	0.7506	0.3030	0.3279	0.054*	
C17	0.7611 (6)	0.2329 (6)	0.4454 (4)	0.0467 (18)	
O17	0.7546 (5)	0.1949 (5)	0.5001 (3)	0.0697 (19)	
C11	0.8673 (5)	0.2557 (6)	0.4170 (4)	0.0386 (15)	
C12	0.9137 (6)	0.3478 (5)	0.4227 (3)	0.0397 (16)	
I12	0.83463 (5)	0.46721 (5)	0.46533 (3)	0.0580 (2)	
C13	1.0151 (6)	0.3627 (6)	0.4001 (4)	0.0489 (19)	
H13	1.0461	0.4246	0.4041	0.059*	
C14	1.0693 (7)	0.2870 (8)	0.3723 (5)	0.064 (3)	
H14	1.1371	0.2976	0.3571	0.077*	
C15	1.0243 (8)	0.1942 (9)	0.3665 (5)	0.075 (3)	
H15	1.0614	0.1423	0.3476	0.090*	
C16	0.9247 (7)	0.1806 (7)	0.3889 (5)	0.063 (2)	
H16	0.8945	0.1184	0.3851	0.076*	
C41	0.4211 (5)	0.4122 (5)	0.3293 (3)	0.0362 (15)	
C42	0.3441 (6)	0.4490 (6)	0.3699 (4)	0.0471 (19)	
H42	0.3446	0.4326	0.4145	0.057*	
C43	0.2655 (6)	0.5101 (6)	0.3459 (4)	0.0446 (18)	
H43	0.2138	0.5329	0.3744	0.053*	
C44	0.2632 (6)	0.5375 (6)	0.2800 (4)	0.0427 (16)	
C45	0.3388 (6)	0.5001 (6)	0.2392 (3)	0.0473 (19)	
H45	0.3379	0.5169	0.1947	0.057*	
C46	0.4155 (6)	0.4388 (6)	0.2622 (4)	0.0471 (19)	
H46	0.4651	0.4141	0.2329	0.056*	
O44	0.1910 (5)	0.5988 (5)	0.2516 (3)	0.0615 (16)	
C441	0.1209 (8)	0.6484 (8)	0.2934 (5)	0.078 (3)	
H41A	0.0746	0.6886	0.2674	0.117*	
H41B	0.1595	0.6896	0.3234	0.117*	
H41C	0.0807	0.6011	0.3179	0.117*	

### 1-(2-Iodobenzoyl)-4-(4-methoxyphenyl)piperazine (V) Atomic displacement parameters (Å^2^)

**Table:**

	*U*^11^	*U*^22^	*U*^33^	*U*^12^	*U*^13^	*U*^23^
N1	0.036 (3)	0.044 (3)	0.053 (4)	0.000 (3)	−0.004 (3)	0.022 (3)
C2	0.037 (4)	0.076 (6)	0.070 (6)	−0.001 (4)	−0.004 (4)	0.037 (5)
C3	0.037 (4)	0.071 (6)	0.048 (4)	−0.001 (4)	0.006 (3)	0.029 (4)
N4	0.030 (3)	0.045 (3)	0.042 (3)	0.000 (3)	0.000 (2)	0.013 (3)
C5	0.034 (4)	0.060 (5)	0.040 (4)	0.002 (3)	0.000 (3)	0.017 (4)
C6	0.037 (4)	0.055 (5)	0.044 (4)	0.005 (3)	−0.001 (3)	0.010 (4)
C17	0.037 (4)	0.046 (4)	0.057 (5)	−0.006 (3)	−0.009 (4)	0.012 (4)
O17	0.048 (3)	0.094 (5)	0.067 (4)	−0.001 (3)	−0.008 (3)	0.039 (4)
C11	0.034 (3)	0.041 (4)	0.041 (4)	0.000 (3)	−0.008 (3)	0.003 (3)
C12	0.041 (4)	0.042 (4)	0.036 (4)	0.003 (3)	−0.009 (3)	−0.001 (3)
I12	0.0702 (4)	0.0501 (3)	0.0538 (3)	0.0045 (3)	−0.0031 (3)	−0.0162 (3)
C13	0.047 (4)	0.056 (5)	0.044 (4)	−0.012 (4)	−0.007 (4)	−0.001 (4)
C14	0.036 (4)	0.098 (8)	0.059 (5)	0.005 (5)	0.003 (4)	0.001 (5)
C15	0.056 (6)	0.088 (8)	0.081 (7)	0.036 (6)	−0.007 (5)	−0.017 (6)
C16	0.055 (5)	0.057 (6)	0.077 (6)	0.004 (4)	−0.012 (5)	−0.008 (5)
C41	0.031 (3)	0.038 (4)	0.040 (4)	−0.002 (3)	−0.002 (3)	0.000 (3)
C42	0.041 (4)	0.061 (5)	0.039 (4)	0.003 (4)	0.008 (3)	0.015 (4)
C43	0.036 (4)	0.044 (4)	0.053 (4)	0.003 (3)	0.010 (3)	0.006 (3)
C44	0.036 (4)	0.043 (4)	0.049 (4)	0.003 (3)	−0.005 (3)	0.002 (3)
C45	0.059 (5)	0.054 (5)	0.030 (3)	0.012 (4)	−0.006 (3)	0.002 (3)
C46	0.048 (4)	0.055 (5)	0.038 (4)	0.017 (4)	0.002 (3)	−0.003 (3)
O44	0.057 (4)	0.066 (4)	0.062 (4)	0.026 (3)	−0.004 (3)	0.008 (3)
C441	0.062 (6)	0.084 (7)	0.089 (7)	0.032 (6)	−0.001 (6)	−0.001 (6)

### 1-(2-Iodobenzoyl)-4-(4-methoxyphenyl)piperazine (V) Geometric parameters (Å, º)

**Table:**

N1—C17	1.342 (9)	C13—C14	1.358 (12)
N1—C2	1.452 (9)	C13—H13	0.9300
N1—C6	1.453 (9)	C14—C15	1.387 (14)
C2—C3	1.490 (11)	C14—H14	0.9300
C2—H2A	0.9700	C15—C16	1.362 (13)
C2—H2B	0.9700	C15—H15	0.9300
C3—N4	1.471 (9)	C16—H16	0.9300
C3—H3A	0.9700	C41—C42	1.375 (10)
C3—H3B	0.9700	C41—C46	1.407 (10)
N4—C41	1.406 (9)	C42—C43	1.388 (10)
N4—C5	1.464 (8)	C42—H42	0.9300
C5—C6	1.512 (10)	C43—C44	1.386 (10)
C5—H5A	0.9700	C43—H43	0.9300
C5—H5B	0.9700	C44—O44	1.368 (9)
C6—H6A	0.9700	C44—C45	1.368 (10)
C6—H6B	0.9700	C45—C46	1.365 (10)
C17—O17	1.224 (9)	C45—H45	0.9300
C17—C11	1.505 (10)	C46—H46	0.9300
C11—C16	1.377 (11)	O44—C441	1.403 (10)
C11—C12	1.385 (10)	C441—H41A	0.9600
C12—C13	1.389 (10)	C441—H41B	0.9600
C12—I12	2.093 (7)	C441—H41C	0.9600
			
C17—N1—C2	120.8 (6)	C13—C12—I12	118.2 (6)
C17—N1—C6	125.0 (6)	C14—C13—C12	120.1 (8)
C2—N1—C6	114.0 (6)	C14—C13—H13	119.9
N1—C2—C3	112.1 (7)	C12—C13—H13	119.9
N1—C2—H2A	109.2	C13—C14—C15	120.5 (8)
C3—C2—H2A	109.2	C13—C14—H14	119.7
N1—C2—H2B	109.2	C15—C14—H14	119.7
C3—C2—H2B	109.2	C16—C15—C14	118.7 (9)
H2A—C2—H2B	107.9	C16—C15—H15	120.6
N4—C3—C2	110.3 (7)	C14—C15—H15	120.6
N4—C3—H3A	109.6	C15—C16—C11	122.3 (9)
C2—C3—H3A	109.6	C15—C16—H16	118.8
N4—C3—H3B	109.6	C11—C16—H16	118.8
C2—C3—H3B	109.6	C42—C41—N4	123.9 (6)
H3A—C3—H3B	108.1	C42—C41—C46	116.6 (7)
C41—N4—C5	116.5 (6)	N4—C41—C46	119.4 (6)
C41—N4—C3	116.5 (6)	C41—C42—C43	121.6 (7)
C5—N4—C3	109.0 (6)	C41—C42—H42	119.2
N4—C5—C6	110.8 (6)	C43—C42—H42	119.2
N4—C5—H5A	109.5	C44—C43—C42	120.8 (7)
C6—C5—H5A	109.5	C44—C43—H43	119.6
N4—C5—H5B	109.5	C42—C43—H43	119.6
C6—C5—H5B	109.5	O44—C44—C45	116.5 (7)
H5A—C5—H5B	108.1	O44—C44—C43	125.6 (7)
N1—C6—C5	110.1 (6)	C45—C44—C43	117.9 (7)
N1—C6—H6A	109.6	C46—C45—C44	121.7 (7)
C5—C6—H6A	109.6	C46—C45—H45	119.2
N1—C6—H6B	109.6	C44—C45—H45	119.2
C5—C6—H6B	109.6	C45—C46—C41	121.4 (7)
H6A—C6—H6B	108.2	C45—C46—H46	119.3
O17—C17—N1	122.6 (7)	C41—C46—H46	119.3
O17—C17—C11	119.6 (7)	C44—O44—C441	117.8 (7)
N1—C17—C11	117.8 (6)	O44—C441—H41A	109.5
C16—C11—C12	118.2 (7)	O44—C441—H41B	109.5
C16—C11—C17	119.1 (7)	H41A—C441—H41B	109.5
C12—C11—C17	122.6 (7)	O44—C441—H41C	109.5
C11—C12—C13	120.1 (7)	H41A—C441—H41C	109.5
C11—C12—I12	121.6 (5)	H41B—C441—H41C	109.5
			
C17—N1—C2—C3	133.1 (8)	I12—C12—C13—C14	179.8 (6)
C6—N1—C2—C3	−51.4 (11)	C12—C13—C14—C15	−0.3 (13)
N1—C2—C3—N4	54.7 (10)	C13—C14—C15—C16	0.3 (15)
C2—C3—N4—C41	166.2 (6)	C14—C15—C16—C11	0.1 (15)
C2—C3—N4—C5	−59.6 (9)	C12—C11—C16—C15	−0.4 (13)
C41—N4—C5—C6	−165.0 (6)	C17—C11—C16—C15	−175.2 (8)
C3—N4—C5—C6	60.7 (8)	C5—N4—C41—C42	−133.4 (8)
C17—N1—C6—C5	−133.5 (8)	C3—N4—C41—C42	−2.6 (10)
C2—N1—C6—C5	51.2 (9)	C5—N4—C41—C46	49.2 (10)
N4—C5—C6—N1	−56.1 (8)	C3—N4—C41—C46	−180.0 (7)
C2—N1—C17—O17	4.4 (13)	N4—C41—C42—C43	−178.1 (7)
C6—N1—C17—O17	−170.6 (8)	C46—C41—C42—C43	−0.6 (12)
C2—N1—C17—C11	−177.2 (8)	C41—C42—C43—C44	−1.1 (12)
C6—N1—C17—C11	7.8 (12)	C42—C43—C44—O44	−178.5 (7)
O17—C17—C11—C16	79.4 (11)	C42—C43—C44—C45	1.9 (12)
N1—C17—C11—C16	−99.0 (9)	O44—C44—C45—C46	179.5 (7)
O17—C17—C11—C12	−95.1 (10)	C43—C44—C45—C46	−0.9 (12)
N1—C17—C11—C12	86.5 (9)	C44—C45—C46—C41	−0.8 (13)
C16—C11—C12—C13	0.4 (11)	C42—C41—C46—C45	1.6 (12)
C17—C11—C12—C13	174.9 (7)	N4—C41—C46—C45	179.2 (7)
C16—C11—C12—I12	−179.4 (6)	C45—C44—O44—C441	−171.8 (8)
C17—C11—C12—I12	−4.9 (9)	C43—C44—O44—C441	8.6 (13)
C11—C12—C13—C14	0.0 (11)		

### 1-(2-Iodobenzoyl)-4-(4-methoxyphenyl)piperazine (V) Hydrogen-bond geometry (Å, º)

**Table:**

*D*—H···*A*	*D*—H	H···*A*	*D*···*A*	*D*—H···*A*
C3—H3*A*···O17^i^	0.97	2.60	3.542 (10)	164
C2—H2*A*···*Cg*1^i^	0.97	2.87	3.719 (11)	147
C15—H15···*Cg*2^ii^	0.93	2.73	3.656 (12)	172

Symmetry codes: (i) *x*−1/2, −*y*+1/2, −*z*+1; (ii) −*x*+3/2, *y*−1/2, *z*.

### 1-(2-Hydroxybenzoyl)-4-(4-methoxyphenyl)piperazine (VI) Crystal data

**Table:**

C_18_H_20_N_2_O_3_	*D*_x_ = 1.329 Mg m^−^^3^
*M**_r_* = 312.36	Mo *K*α radiation, λ = 0.71073 Å
Orthorhombic, *P**b**c**a*	Cell parameters from 3474 reflections
*a* = 9.7265 (6) Å	θ = 2.7–27.9°
*b* = 12.9084 (9) Å	µ = 0.09 mm^−^^1^
*c* = 24.861 (1) Å	*T* = 293 K
*V* = 3121.4 (3) Å^3^	Plate, yellow
*Z* = 8	0.50 × 0.40 × 0.16 mm
*F*(000) = 1328	

### 1-(2-Hydroxybenzoyl)-4-(4-methoxyphenyl)piperazine (VI) Data collection

**Table:**

Oxford Diffraction Xcalibur diffractometer with Sapphire CCD	3474 independent reflections
Radiation source: Enhance (Mo) X-ray Source	2492 reflections with *I* > 2σ(*I*)
Graphite monochromator	*R*_int_ = 0.020
ω scans	θ_max_ = 27.9°, θ_min_ = 2.7°
Absorption correction: multi-scan (CrysAlis RED; Oxford Diffraction, 2009)	*h* = −12→5
*T*_min_ = 0.917, *T*_max_ = 0.986	*k* = −13→16
11981 measured reflections	*l* = −31→31

### 1-(2-Hydroxybenzoyl)-4-(4-methoxyphenyl)piperazine (VI) Refinement

**Table:**

Refinement on *F*^2^	Primary atom site location: difference Fourier map
Least-squares matrix: full	Hydrogen site location: mixed
*R*[*F*^2^ > 2σ(*F*^2^)] = 0.041	H atoms treated by a mixture of independent and constrained refinement
*wR*(*F*^2^) = 0.100	*w* = 1/[σ^2^(*F*_o_^2^) + (0.038*P*)^2^ + 0.9467*P*] where *P* = (*F*_o_^2^ + 2*F*_c_^2^)/3
*S* = 1.04	(Δ/σ)_max_ < 0.001
3474 reflections	Δρ_max_ = 0.16 e Å^−^^3^
212 parameters	Δρ_min_ = −0.17 e Å^−^^3^
0 restraints	

### 1-(2-Hydroxybenzoyl)-4-(4-methoxyphenyl)piperazine (VI) Special details

**Table:**

Geometry. All esds (except the esd in the dihedral angle between two l.s. planes) are estimated using the full covariance matrix. The cell esds are taken into account individually in the estimation of esds in distances, angles and torsion angles; correlations between esds in cell parameters are only used when they are defined by crystal symmetry. An approximate (isotropic) treatment of cell esds is used for estimating esds involving l.s. planes.

### 1-(2-Hydroxybenzoyl)-4-(4-methoxyphenyl)piperazine (VI) Fractional atomic coordinates and isotropic or equivalent isotropic displacement parameters (Å^2^)

**Table:**

	*x*	*y*	*z*	*U*_iso_*/*U*_eq_	
N1	0.65619 (12)	0.42451 (10)	0.35751 (5)	0.0368 (3)	
C2	0.64088 (15)	0.36047 (14)	0.40542 (6)	0.0448 (4)	
H2A	0.5498	0.3298	0.4057	0.054*	
H2B	0.6498	0.4035	0.4372	0.054*	
C3	0.74781 (15)	0.27547 (13)	0.40711 (6)	0.0432 (4)	
H3A	0.7390	0.2367	0.4404	0.052*	
H3B	0.7334	0.2281	0.3774	0.052*	
N4	0.88519 (12)	0.32027 (10)	0.40359 (5)	0.0384 (3)	
C5	0.89929 (15)	0.38044 (13)	0.35376 (6)	0.0429 (4)	
H5A	0.8865	0.3351	0.3230	0.051*	
H5B	0.9912	0.4094	0.3517	0.051*	
C6	0.79474 (14)	0.46694 (12)	0.35178 (6)	0.0401 (4)	
H6A	0.8126	0.5159	0.3806	0.048*	
H6B	0.8023	0.5035	0.3178	0.048*	
C17	0.54733 (14)	0.44756 (11)	0.32643 (5)	0.0324 (3)	
O17	0.43132 (10)	0.41265 (8)	0.33693 (4)	0.0404 (3)	
C11	0.56584 (14)	0.52022 (11)	0.28009 (6)	0.0329 (3)	
C12	0.64738 (14)	0.49883 (11)	0.23528 (6)	0.0349 (3)	
O12	0.71754 (12)	0.40749 (9)	0.23434 (4)	0.0461 (3)	
H12	0.789 (2)	0.4130 (15)	0.2100 (8)	0.069*	
C13	0.65334 (16)	0.56961 (12)	0.19321 (6)	0.0412 (4)	
H13	0.7060	0.5548	0.1629	0.049*	
C14	0.58174 (17)	0.66145 (13)	0.19606 (6)	0.0471 (4)	
H14	0.5888	0.7092	0.1682	0.057*	
C15	0.49942 (17)	0.68344 (13)	0.23996 (7)	0.0483 (4)	
H15	0.4501	0.7451	0.2415	0.058*	
C16	0.49147 (15)	0.61263 (12)	0.28139 (6)	0.0408 (4)	
H16	0.4354	0.6268	0.3108	0.049*	
C41	0.99766 (14)	0.25442 (12)	0.41654 (6)	0.0355 (3)	
C42	0.98274 (16)	0.15034 (13)	0.42966 (6)	0.0419 (4)	
H42	0.8956	0.1207	0.4291	0.050*	
C43	1.09521 (16)	0.08983 (13)	0.44351 (6)	0.0450 (4)	
H43	1.0825	0.0204	0.4523	0.054*	
C44	1.22554 (15)	0.13157 (13)	0.44442 (6)	0.0407 (4)	
C45	1.24231 (15)	0.23528 (13)	0.43236 (7)	0.0451 (4)	
H45	1.3295	0.2648	0.4335	0.054*	
C46	1.13003 (15)	0.29544 (13)	0.41862 (7)	0.0446 (4)	
H46	1.1432	0.3651	0.4106	0.054*	
O44	1.33074 (12)	0.06448 (10)	0.45724 (5)	0.0569 (3)	
C441	1.46702 (17)	0.10399 (16)	0.45482 (7)	0.0576 (5)	
H41A	1.5311	0.0492	0.4622	0.086*	
H41B	1.4778	0.1579	0.4811	0.086*	
H41C	1.4842	0.1315	0.4196	0.086*	

### 1-(2-Hydroxybenzoyl)-4-(4-methoxyphenyl)piperazine (VI) Atomic displacement parameters (Å^2^)

**Table:**

	*U*^11^	*U*^22^	*U*^33^	*U*^12^	*U*^13^	*U*^23^
N1	0.0309 (6)	0.0442 (7)	0.0354 (6)	−0.0022 (5)	−0.0010 (5)	0.0077 (6)
C2	0.0336 (8)	0.0634 (11)	0.0374 (8)	0.0013 (7)	0.0034 (6)	0.0138 (8)
C3	0.0354 (8)	0.0504 (10)	0.0437 (8)	−0.0035 (7)	0.0016 (7)	0.0152 (7)
N4	0.0308 (6)	0.0418 (7)	0.0425 (7)	−0.0002 (5)	0.0028 (5)	0.0091 (6)
C5	0.0340 (7)	0.0480 (10)	0.0466 (9)	−0.0019 (7)	0.0044 (6)	0.0096 (7)
C6	0.0340 (8)	0.0407 (9)	0.0454 (8)	−0.0053 (6)	−0.0044 (6)	0.0076 (7)
C17	0.0336 (7)	0.0309 (8)	0.0327 (7)	0.0007 (6)	0.0008 (6)	−0.0043 (6)
O17	0.0318 (5)	0.0467 (6)	0.0428 (6)	−0.0038 (4)	−0.0005 (4)	0.0058 (5)
C11	0.0317 (7)	0.0342 (8)	0.0328 (7)	−0.0023 (6)	−0.0033 (6)	0.0008 (6)
C12	0.0340 (7)	0.0356 (8)	0.0352 (8)	−0.0029 (6)	−0.0019 (6)	−0.0021 (6)
O12	0.0488 (7)	0.0439 (7)	0.0456 (6)	0.0073 (5)	0.0128 (5)	0.0021 (5)
C13	0.0437 (9)	0.0464 (9)	0.0334 (8)	−0.0071 (7)	0.0001 (6)	0.0016 (7)
C14	0.0597 (10)	0.0429 (9)	0.0388 (8)	−0.0052 (8)	−0.0061 (7)	0.0100 (7)
C15	0.0574 (10)	0.0369 (9)	0.0507 (10)	0.0068 (7)	−0.0067 (8)	0.0035 (7)
C16	0.0414 (8)	0.0408 (9)	0.0402 (8)	0.0029 (7)	0.0008 (7)	−0.0020 (7)
C41	0.0339 (7)	0.0398 (8)	0.0328 (7)	0.0008 (6)	0.0017 (6)	−0.0008 (6)
C42	0.0386 (8)	0.0441 (9)	0.0430 (8)	−0.0059 (7)	−0.0040 (7)	0.0038 (7)
C43	0.0512 (9)	0.0375 (9)	0.0464 (9)	−0.0011 (7)	−0.0037 (7)	0.0073 (7)
C44	0.0407 (8)	0.0472 (10)	0.0343 (7)	0.0075 (7)	0.0020 (6)	0.0006 (7)
C45	0.0316 (7)	0.0491 (10)	0.0545 (10)	−0.0004 (7)	0.0036 (7)	0.0016 (8)
C46	0.0390 (8)	0.0359 (8)	0.0589 (10)	−0.0003 (7)	0.0040 (7)	0.0032 (8)
O44	0.0447 (7)	0.0560 (8)	0.0700 (8)	0.0120 (6)	−0.0018 (6)	0.0148 (6)
C441	0.0427 (10)	0.0790 (14)	0.0509 (10)	0.0160 (9)	0.0008 (7)	0.0129 (9)

### 1-(2-Hydroxybenzoyl)-4-(4-methoxyphenyl)piperazine (VI) Geometric parameters (Å, º)

**Table:**

N1—C17	1.3440 (18)	C13—C14	1.377 (2)
N1—C2	1.4575 (18)	C13—H13	0.9300
N1—C6	1.4616 (18)	C14—C15	1.383 (2)
C2—C3	1.512 (2)	C14—H14	0.9300
C2—H2A	0.9700	C15—C16	1.379 (2)
C2—H2B	0.9700	C15—H15	0.9300
C3—N4	1.4587 (18)	C16—H16	0.9300
C3—H3A	0.9700	C41—C42	1.390 (2)
C3—H3B	0.9700	C41—C46	1.393 (2)
N4—C41	1.4222 (18)	C42—C43	1.388 (2)
N4—C5	1.4686 (19)	C42—H42	0.9300
C5—C6	1.511 (2)	C43—C44	1.378 (2)
C5—H5A	0.9700	C43—H43	0.9300
C5—H5B	0.9700	C44—O44	1.3779 (18)
C6—H6A	0.9700	C44—C45	1.382 (2)
C6—H6B	0.9700	C45—C46	1.383 (2)
C17—O17	1.2428 (16)	C45—H45	0.9300
C17—C11	1.496 (2)	C46—H46	0.9300
C11—C12	1.395 (2)	O44—C441	1.422 (2)
C11—C16	1.395 (2)	C441—H41A	0.9600
C12—O12	1.3625 (18)	C441—H41B	0.9600
C12—C13	1.390 (2)	C441—H41C	0.9600
O12—H12	0.92 (2)		
			
C17—N1—C2	120.99 (12)	C12—O12—H12	108.7 (12)
C17—N1—C6	125.97 (12)	C14—C13—C12	120.40 (14)
C2—N1—C6	112.73 (11)	C14—C13—H13	119.8
N1—C2—C3	111.34 (12)	C12—C13—H13	119.8
N1—C2—H2A	109.4	C13—C14—C15	120.69 (15)
C3—C2—H2A	109.4	C13—C14—H14	119.7
N1—C2—H2B	109.4	C15—C14—H14	119.7
C3—C2—H2B	109.4	C16—C15—C14	119.06 (15)
H2A—C2—H2B	108.0	C16—C15—H15	120.5
N4—C3—C2	109.92 (13)	C14—C15—H15	120.5
N4—C3—H3A	109.7	C15—C16—C11	121.35 (14)
C2—C3—H3A	109.7	C15—C16—H16	119.3
N4—C3—H3B	109.7	C11—C16—H16	119.3
C2—C3—H3B	109.7	C42—C41—C46	117.07 (14)
H3A—C3—H3B	108.2	C42—C41—N4	123.40 (13)
C41—N4—C3	117.01 (12)	C46—C41—N4	119.48 (14)
C41—N4—C5	115.80 (11)	C43—C42—C41	121.33 (14)
C3—N4—C5	110.23 (11)	C43—C42—H42	119.3
N4—C5—C6	110.83 (12)	C41—C42—H42	119.3
N4—C5—H5A	109.5	C44—C43—C42	120.62 (15)
C6—C5—H5A	109.5	C44—C43—H43	119.7
N4—C5—H5B	109.5	C42—C43—H43	119.7
C6—C5—H5B	109.5	C43—C44—O44	116.19 (15)
H5A—C5—H5B	108.1	C43—C44—C45	118.95 (14)
N1—C6—C5	109.90 (13)	O44—C44—C45	124.86 (14)
N1—C6—H6A	109.7	C44—C45—C46	120.29 (15)
C5—C6—H6A	109.7	C44—C45—H45	119.9
N1—C6—H6B	109.7	C46—C45—H45	119.9
C5—C6—H6B	109.7	C45—C46—C41	121.71 (15)
H6A—C6—H6B	108.2	C45—C46—H46	119.1
O17—C17—N1	120.95 (13)	C41—C46—H46	119.1
O17—C17—C11	119.87 (12)	C44—O44—C441	117.20 (14)
N1—C17—C11	119.11 (12)	O44—C441—H41A	109.5
C12—C11—C16	118.83 (13)	O44—C441—H41B	109.5
C12—C11—C17	123.99 (13)	H41A—C441—H41B	109.5
C16—C11—C17	117.11 (12)	O44—C441—H41C	109.5
O12—C12—C13	122.33 (13)	H41A—C441—H41C	109.5
O12—C12—C11	118.02 (13)	H41B—C441—H41C	109.5
C13—C12—C11	119.64 (14)		
			
C17—N1—C2—C3	131.68 (15)	C11—C12—C13—C14	1.4 (2)
C6—N1—C2—C3	−54.39 (18)	C12—C13—C14—C15	−2.0 (2)
N1—C2—C3—N4	55.85 (17)	C13—C14—C15—C16	0.9 (2)
C2—C3—N4—C41	166.38 (12)	C14—C15—C16—C11	0.7 (2)
C2—C3—N4—C5	−58.51 (16)	C12—C11—C16—C15	−1.3 (2)
C41—N4—C5—C6	−164.75 (13)	C17—C11—C16—C15	−178.57 (14)
C3—N4—C5—C6	59.54 (17)	C3—N4—C41—C42	3.0 (2)
C17—N1—C6—C5	−132.34 (15)	C5—N4—C41—C42	−129.62 (15)
C2—N1—C6—C5	54.10 (17)	C3—N4—C41—C46	−174.21 (14)
N4—C5—C6—N1	−56.31 (17)	C5—N4—C41—C46	53.14 (19)
C2—N1—C17—O17	−1.9 (2)	C46—C41—C42—C43	−0.8 (2)
C6—N1—C17—O17	−174.98 (14)	N4—C41—C42—C43	−178.14 (14)
C2—N1—C17—C11	175.29 (13)	C41—C42—C43—C44	−0.2 (2)
C6—N1—C17—C11	2.2 (2)	C42—C43—C44—O44	−178.28 (14)
O17—C17—C11—C12	−117.64 (16)	C42—C43—C44—C45	1.3 (2)
N1—C17—C11—C12	65.13 (19)	C43—C44—C45—C46	−1.2 (2)
O17—C17—C11—C16	59.44 (18)	O44—C44—C45—C46	178.31 (15)
N1—C17—C11—C16	−117.79 (15)	C44—C45—C46—C41	0.1 (2)
C16—C11—C12—O12	−178.98 (13)	C42—C41—C46—C45	0.9 (2)
C17—C11—C12—O12	−1.9 (2)	N4—C41—C46—C45	178.32 (14)
C16—C11—C12—C13	0.2 (2)	C43—C44—O44—C441	175.77 (14)
C17—C11—C12—C13	177.27 (13)	C45—C44—O44—C441	−3.8 (2)
O12—C12—C13—C14	−179.40 (14)		

### 1-(2-Hydroxybenzoyl)-4-(4-methoxyphenyl)piperazine (VI) Hydrogen-bond geometry (Å, º)

**Table:**

*D*—H···*A*	*D*—H	H···*A*	*D*···*A*	*D*—H···*A*
O12—H12···O17^i^	0.92 (2)	1.81 (2)	2.7327 (15)	175.4 (18)

Symmetry code: (i) *x*+1/2, *y*, −*z*+1/2.
